# Boosting cadmium tolerance in *Phoebe zhennan*: the synergistic effects of exogenous nitrogen and phosphorus treatments promoting antioxidant defense and root development

**DOI:** 10.3389/fpls.2024.1340287

**Published:** 2024-02-01

**Authors:** Juan Zhang, Noman Shoaib, Kexin Lin, Nishbah Mughal, Xiaogang Wu, Xiaoming Sun, Lin Zhang, Kaiwen Pan

**Affiliations:** ^1^ CAS Key Laboratory of Mountain Ecological Restoration and Bioresource Utilization & Ecological Restoration Biodiversity Conservation Key Laboratory of Sichuan Province, Chengdu Institute of Biology, Chinese Academy of Sciences, University of Chinese Academy of Sciences, Beijing, China; ^2^ College of Agronomy, Sichuan Agricultural University, Chengdu, Sichuan, China

**Keywords:** cadmium toxicity, root orders, antioxidants, glutathione synthetase, nutrient uptake

## Abstract

Plants possess intricate defense mechanisms to resist cadmium (Cd) stress, including strategies like metal exclusion, chelation, osmoprotection, and the regulation of photosynthesis, with antioxidants playing a pivotal role. The application of nitrogen (N) and phosphorus (P) fertilizers are reported to bolster these defenses against Cd stress. Several studies investigated the effects of N or P on Cd stress in non-woody plants and crops. However, the relationship between N, P application, and Cd stress resistance in valuable timber trees remains largely unexplored. This study delves into the Cd tolerance mechanisms of *Phoebe zhennan*, a forest tree species, under various treatments: Cd exposure alone, combined Cd stress with either N or P and Cd stress with both N and P application. Our results revealed that the P application enhanced root biomass and facilitated the translocation of essential nutrients like K, Mn, and Zn. Conversely, N application, especially under Cd stress, significantly inhibited plant growth, with marked reductions in leaf and stem biomass. Additionally, while the application of P resulted in reduced antioxidant enzyme levels, the combined application of N and P markedly amplified the activities of peroxidase by 266.36%, superoxide dismutase by 168.44%, and ascorbate peroxidase by 26.58% under Cd stress. This indicates an amplified capacity of the plant to neutralize reactive oxygen species. The combined treatment also led to effective regulation of nutrient and Cd distribution in roots, shoots, and leaves, illustrating a synergistic effect in mitigating toxic impact of N. The study also highlights a significant alteration in photosynthetic activities under different treatments. The N addition generally reduced chlorophyll content by over 50%, while P and NP treatments enhanced transpiration rates by up to 58.02%. Our findings suggest P and NP fertilization can manage Cd toxicity by facilitating antioxidant production, osmoprotectant, and root development, thus enhancing Cd tolerance processes, and providing novel strategies for managing Cd contamination in the environment.

## Graphical Abstract

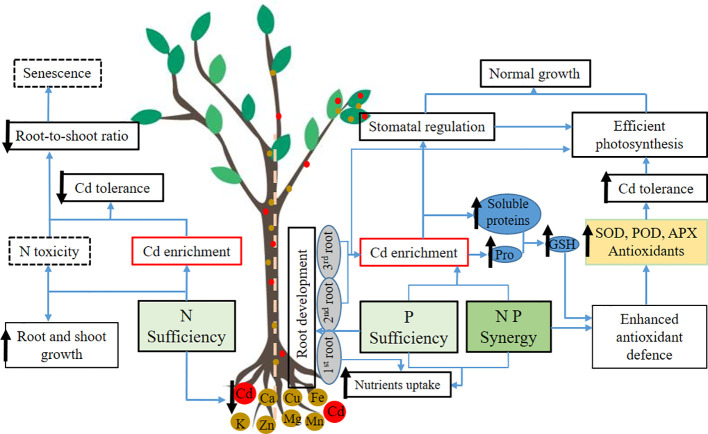

## 1 Introduction

Cadmium (Cd) is a potent heavy metal pollutant that even at low concentrations poses a significant threat to arable land (Li et al., 2017; Shi et al., 2019). Its contamination presents a significant environmental concern due to its considerable biological toxicity, persistence in ecosystems, and propensity to accumulate in organisms including humans. The primary route of Cd exposure to humans is through the consumption of contaminated crops which contribute significantly to daily Cd intake, accounting for more than 70% of total dietary intake (Ismael et al., 2019). This is largely due to the uptake of Cd by plants from contaminated soils, a process that can pose substantial health risks including short-term intestinal disease in those consuming these plants (Guo et al., 2018; Ismael et al., 2019). As such, soils with high Cd concentrations are unsuitable for cultivating edible crops. This has prompted interest in identifying and planting non-edible economically valuable plants, such as certain tree species in Cd-contaminated soils.

Heavy metals in soil environments are typically stabilized by sorption, precipitation, and complexation with soil constituents, thus reducing their bioavailability to plants (Seshadri et al., 2016). However, certain elements have been found to enhance the bioavailable fraction of these heavy metals, for example, altering the chemical forms of Cd to increase its bioavailability (Dong et al., 2007; Yin et al., 2016). This potentially heightened Cd stress levels in the presence of elements like zinc (Zn), iron (Fe), selenium (Se), nitrogen (N), and phosphorus (P). Zn and Cd, due to their chemical similarities, can either inhibit or enhance each other’s uptake by plants through competitive interactions (Balen et al., 2011). Fe affects Cd availability in soil through redox reactions; oxidized Fe binds Cd, reducing its bioavailability, while reduced Fe under conditions like waterlogging can increase Cd availability (Zhang et al., 2012). Se forms complexes with Cd, reducing its bioavailability and mitigating Cd toxicity in plants (Riaz et al., 2021). The N and P are vital macronutrients that underpin plant growth and development, with their fertilizers enhancing soil nutrients efficiency and plant productivity across diverse ecosystems (Reich et al., 2003).

Plants have developed a variety of molecular strategies to tolerate and accumulate Cd. This included generating antioxidants and regulating enzymes like superoxide dismutase (SOD), which neutralized superoxide radicals (O_2_^·−^), turning them into hydrogen peroxide (H_2_O_2_) and oxygen (Meyer et al., 2015). Other essential enzymes, such as peroxidase (POD) and catalase (CAT), played a role in transforming H_2_O_2_ and O_2_^·−^ into water. The amino acid proline had been known to boost antioxidant systems and counteract reactive oxygen species (ROS) during oxidative stress (El Rasafi et al., 2022). Although there are multiple strategies to reduce Cd accumulation in crops, many proved to be impracticable due to issues like cost, duration, and interference with farming operations (Verma et al., 2021). Fertilizer management is considered the most economical and effective approach to decrease Cd accumulation (Zhen et al., 2021). Past studies indicated that N fertilizers (nitrate; NO_3_^–^-N, ammonium; NH_4_^+^-N) compounds could influence accumulation of Cd in plant tissues. Lower N levels enhanced soil Cd bioavailability, countered the limitations on nutrient absorption caused by Cd stress, and diminished Cd absorption (Tan et al., 2017). Using NH_4_^+^-N had shown to counteract the growth-deterrent effects of elevated Cd concentrations (Lin et al., 2011). In a similar vein, P fertilizers improved plant resistance to abiotic stress by decreasing phospholipid oxidation and impacting Cd absorption and movement (Tariq et al., 2017; Dai et al., 2018). Importantly, N and P boosted chlorophyll formation and photosynthesis, fortifying plant resistance to environmental challenges (Tan et al., 2017; Jia et al., 2020). Even with pivotal roles of N and P in plant growth, there remained a gap in understanding their effects on plant physiological and ecological functions under heavy metal stress. Earlier studies mainly centered on the roles of N or P in physio-biochemical adjustments under Cd stress, predominantly in non-woody plants and crops (Ismael et al., 2019; Yoshida et al., 2019; Raza et al., 2020; Zhou et al., 2020).

Woody plants exhibit diverse preferences for fertilizer absorption, influenced by factors such as species, soil characteristics, pH, temperature, and the nature of plant roots (He et al., 2014; Tariq et al., 2019). The root system of trees presents a refined, tiered structure. Here, the lower-order roots are mainly responsible for water and nutrient absorption, whereas the higher-order roots prioritize water transportation (Kong et al., 2010). The primary route for Cd absorption in plants is through their root system (Dai et al., 2018), which consequently faces the adverse impacts of these metal ions (Tariq et al., 2017). When subjected to heavy metal stress, the root system undergoes morphological and physiological adaptations (Tariq et al., 2017; Liu et al., 2020), reflecting its immediate response or adjustment to the nutrients and Cd present in the soil. A primary response observed was the modification in root architecture (Bochicchio et al., 2015). Plants often reduced their primary root growth while enhancing the growth and branching of lateral roots (Waidmann et al., 2020). This adaptation allowed them to explore a broader soil volume without penetrating deeper into potentially contaminated layers. Additionally, Roots have been observed to develop a thicker epidermis and release specific compounds that bind to heavy metals, thereby reducing their absorption (Emamverdian et al., 2015). On a physiological level, roots amplify the production of metal-binding proteins and antioxidants (Feki et al., 2021). These proteins capture heavy metals, shielding cells from harm. Meanwhile, antioxidants counteract the oxidative stress that these metals introduce within plant cells (Bhaduri and Fulekar, 2012). However, despite comprehensive studies on the physiological and morphological responses of root orders, there is a notable gap in research exploring the impact of N and P additions on root development and structure under Cd stress. This oversight is especially evident when examining the reactions of different root orders, underscoring a significant research void.

*Phoebe zhennan*, a unique and nationally protected species in China, is valued for its adaptability, ornamental features, high-quality timber, and cultural importance in traditional Chinese furniture and architecture (Tie et al., 2019; Tariq et al., 2022). Despite its economic importance, *P. zhennan* is classified as threatened by the International Union for Conservation of Nature (IUCN) and often fails to reach its full growth potential due to factors such as heavy metal contamination (Gao et al., 2016). While studies have investigated *P. zhennan* growth and resistance to Cd stress, it lacks comprehensive insights into the effects of Cd stress on growth and potential physio-biochemical adaptations (Tariq et al., 2017; Tie et al., 2019). However, our understanding of *P. zhennan* metabolic responses to Cd stress and potential morphological and physio-biochemical adaptations remains limited. Furthermore, research on how N and P fertilizers, either separately or in combination, could mitigate Cd stress in *P. zhennan* is non-existent to our knowledge. Hence, research to further our understanding of *P. zhennan* tolerance to Cd stress and the impact of N and P fertilizer addition is both timely and pertinent.

Our hypothesis posits that the application of N and P fertilization, either independently or synergistically, could alleviate the stress induced by Cd in *P.* zhennan, making this species a potential candidate to utilize the Cd-contaminated soils. To address this, we conducted a pot experiment aimed to investigate the combined effects of N and P fertilization on mitigating Cd stress in *P. zhennan* seedlings. This included examining both the physiological and morphological responses under Cd stress and evaluating the role of N and P application in enhancing the tolerance capacity of the plant. We assessed Cd uptake by roots, accumulation in shoots, biochemical changes, and changes in antioxidant enzymatic activities. This study provides a systematic exploration of the interactions between Cd, N, and P, offering valuable insights for the development of N and P management strategies for heavy metal stress tolerance.

## 2 Materials and methods

### 2.1 Experimental design

One-year-old saplings of *P. zhennan* saplings (the root systems of each sapling were carefully removed from the soil), sourced from S. Lee’s gardening farm (103°47’E, 30°55’N), were transplanted into 20-L plastic pots (15 cm inner diameter, 17 cm outer diameter, 30 cm height) containing 10 kg of farm-derived homogenous soil. The soil consisted of 1.78% organic matter, 1.47g·kg^–1^ total N, 10.59g·kg^–1^ total carbon, 0.1233g·kg^–1^ available N, 84.003mg·kg^–1^ available P, 0.1371mg·kg^–1^ Cd, and had a pH of 7.7. The saplings were cultivated in a greenhouse at the Chengdu Institute of Biology, Chinese Academy of Sciences (104°4’E, 30°37’N, 478.5m altitude) beginning on March 1, 2021.

After three months of growth, the plants underwent randomized treatment combinations of two Cd levels (absent and high at 15mg/kg) and nutrient additions (N, P, and NP). Eight treatment conditions were established: CK (no nitrogen, phosphorus, and Cd); N (adding N, no P, and Cd); P (adding P, no N, and Cd); NP (adding N and P, no Cd); CdH (adding high Cd, no N, and P); CdHN (adding high Cd and N, no P); CdHP (adding high Cd and P, no N); and CdHNP (adding high Cd, N, and P), with at least 12 replicates, three biological replicates, and four saplings each. Based on the Cd pollution condition in Sichuan soil and combining the results from Tie, 2021 (Xiang, 2021) which indicated that *P. zhennan* seedlings are not suitable for growth when the soil Cd content exceeds 8 mg/kg, the impact becomes particularly significant when the applied Cd level reaches 20 mg/kg or higher. Therefore, the Cd treatment is set at 15 mg/kg. The Cd was dissolved in the form of CdCl_2_·2.5H_2_O (precipitated pure) solution and applied three times to irrigate the potting soil. The control treatment involved the addition of an equivalent volume of deionized water to the plants. The N fertilizer was applied as ammonium nitrate (NH_4_NO_3_, 34.99% N) at a dose of 60 mg/kg, totalling 600 mg N (He et al., 2015), and P fertilizer is added as sodium dihydrogen phosphate (NaH_2_PO_4_, 25.5% P) at a dose of 60 mg/kg, totalling 600 mg P (Olatunji et al., 2018). For each treatment, reagents are dissolved in 200 mL of water and mixed before being added, divided into three applications, with nutrient addition treatment conducted every 15 days (Tariq et al., 2018).

### 2.2 Morphological parameters

The plants were gathered at the end of the experiments and various measurements were taken. The increase in plant height (IPH) and ground diameter (IGD) were measured or computed. The leaf, stem, and root dry weights (LDW, SDW, and RDW), as well as the aboveground biomass (AB) and total biomass (TB), were weighed for each sapling. The dry weights of the 1st, 2nd, and 3rd roots were dried and weighed. The harvested roots were washed and scanned using a root scanner, and their length (RL), the average diameter (ARD), surface area (RS), and volume (RV) were analyzed using WinRHIZO Pro 2012.

### 2.3 Photosynthesis analysis

To determine the concentrations of Chla (chlorophyll a), Chlb (chlorophyll b), TChl (total chlorophyll), and Caro (carotenoids), leaf tissue was extracted in 80% chilled acetone and quantified at wavelengths of 470, 646, and 663 nm, as described by (Lichtenthaler, 1987). Photosynthetic parameters, including Pn (net photosynthetic rate), Gs (stomatal conductance), Ci (intercellular CO_2_ concentration), and Tr (transpiration rate), were measured using the LI-COR 6400 portable photosynthesis system (LI-6400, LI-COR Inc, USA) between 9:00-11:30 am, according to the method described by (Yang et al., 2010).

### 2.4 Superoxide radicals (O_2_^·−^), relative conductivity, and malondialdehyde

We exclusively utilized leaf samples to determine oxidative stress markers and antioxidant analysis. The leaf tissues were carefully harvested from the plants under study and were immediately processed for the analysis of oxidative stress markers and antioxidant enzyme activities. This choice was made considering that leaves are primary sites of ROS production under metal stress and provide crucial insights into the defense mechanisms of plant. To measure the O_2_^·−^ (superoxide radicals), a method described by (Zhang et al., 2011) was used. Fresh samples were homogenized in 2mL of 65mmol·L^–1^ phosphate buffer (pH 7.8), and the reaction mixture included 1mL of supernatant, 1mL of 65mM phosphate buffer, 1mL of 10mM hydroxylamine hydrochloride, 1mL of 17mM sulphanilamide, and 1mL of 7 mM α-naphthylamine. After incubation at 25°C for 20min, the mixture was extracted with an equal volume of n-butanol, and the absorbance was measured at 530nm, using NaNO_2_^–^ as the standard curve to calculate the O_2_^·−^ activity. The relative conductivity (RC) and MDA (malondialdehyde) were measured following the method described by (Yang et al., 2022).

### 2.5 Antioxidant enzymes activity determination

The POD activity was quantified using the guaiacol colorimetric method at 470 nm following the procedure of Micro Peroxidase Assay Kit (EC 1.11.1.7, Beijing Solarbio Science & Technology Co. Ltd., Beijing, China) according to (Doerge et al., 1997). POD activity was calculated as an absorbance change of 0.005 per minute per unit.

The CAT activity was determined at 240 nm following the procedure of Catalase Activity Assay Kit (EC 1.11.1.6, Beijing Solarbio Science & Technology Co. Ltd., Beijing, China) according to (Johansson and Borg, 1988).

The SOD activity was measured using the reduction of nitroblue tetrazolium (NBT) photoreduction method as described by (Yang and Miao, 2010). The experiment involved creating a reaction mixture with 50 mM Tris-HCl buffer (pH 7.8), 0.1 mM EDTA, and 13.37 mM methionine. To this 5.7 ml mixture, 200 μl of 0.1 mM riboflavin (in 50 mM Tris-HCl and 0.1 mM EDTA, pH 7.8) and 0.1 ml of enzyme source were added. The reaction started when the test tubes were placed under fluorescent lights and stopped after 30 minutes by removing them from the light. Tubes kept in the dark acted as controls. A light-exposed control without protein showed maximum NBT reduction, indicating peak absorbance at 560 nm.

The APX activity was determined by following the method using the spectrophotometer described by (Nakano and Asada, 1981). To prepare the leaf extract, approximately 0.1 g of fresh leaves with veins removed were ground in an ice bath using 1.5 mL of PBS. The mixture was then centrifuged at 8000 r/min for 10 minutes at 4°C, and the resulting supernatant was retained on ice for analysis. For enzyme assays, 0.05 mL of this supernatant was combined with 0.13 mL of distilled water and 2.8 mL of PBS in a cuvette. The enzymatic reaction commenced upon adding 0.02 mL of 200 mmol·L^-1^ H_2_O_2_ solution, and the absorbance at 290 nm was measured immediately, with readings taken every 15 seconds over a 2-minute period.

### 2.6 Non-enzymatic antioxidant content determination

Proline content in fresh leaf samples (~200mg) was quantified using a method described by (Bates et al., 1973). Samples were homogenized with 5mL of 3% sulfosalicylic acid. 1mL of this homogenate was added to acid-acetic ninhydrin reagent and glacial acetic acid, incubated at 100°C for 1 hour, then cooled and subjected to toluene extraction. Absorbance was read at 520nm.

Soluble protein concentration was gauged following (Bradford, 1976) using Coomassie Brilliant Blue G-250. Fresh samples (100mg) were mixed with phosphate buffer (pH 7.8), and protein concentration was inferred from a standard curve with absorbance read at 595nm.

GSH levels in fresh leaves (~100mg) were determined using a kit from Nanjing Jiancheng Bioengineering Institute, following (Yang et al., 2010). Leaves were ground in liquid nitrogen and homogenized with PBS (5mg fresh leaves:50μL PBS) before centrifugation (10,000 rpm, 30 min, 4°C) and assay performance.

### 2.7 Determination of the Cd content

The Cd amounts in the plant samples were identified directly by ICP-MS (Thermo Fisher Scientific, XSeriesll, Germany) as described by (McBride and Spiers, 2001). The Plant tissues were collected and dried at 80-100°C until a constant weight was achieved. The dried tissues were then ground to a fine powder using a mortar and pestle. 0.5 g of the powdered sample was weighed into a digestion tube, and 2 mL of concentrated nitric acid (HNO_3_) was added. The mixture was then heated on a hot plate until the volume was reduced to approximately 1mL, and the solution turned clear. 1mL of hydrogen peroxide (H_2_O_2_) was added, and the mixture was further heated until the solution turned clear again. Deionized water was added to the digestion tube to bring the final volume to 50mL. The solution was mixed well and filtered through a filter paper or membrane filter to remove any insoluble particles. The Cd levels in the filtered solution were measured using an ICP-MS instrument.

The digestion process involving HNO_3_ and H_2_O_2_ is designed to ensure the complete breakdown of plant cellular structures, thereby releasing Cd from all compartments, including the apoplast. This method allows for the solubilization of Cd bound in both the apoplastic and symplastic pathways of the plant tissues. Consequently, the Cd levels measured by ICP-MS represent the total Cd content, encompassing Cd from all cellular compartments.

### 2.8 Determination of fertilizers content

Plant NO_3_^–^N was determined using the direct extraction method. The plant leaves were ground with deionized water to extract the supernatant. From this extraction, 0.1 mL of supernatant was taken and mixed with a 5% salicylic acid-sulphuric acid solution and 8% NaOH. This mixture was then cooled in an ice water bath until it reached room temperature. Afterward, its absorbance was measured at a wavelength of 410 nm.

For NH_4_^+^-N, the ninhydrin colorimetric method was employed. Fresh leaves, with their main veins removed, were weighed to 0.1 g and then finely chopped. To this, 1 mL of 10% acetic acid solution was added, and the mixture was ground into a homogenate. After centrifugation, 0.5 mL of the supernatant was collected and combined with 1.5 mL of distilled water, 3 mL of ninhydrin reagent, and 0.1 mL of 1% ascorbic acid. This solution was then heated in a boiling water bath for 15 minutes. After a cooling period of 15 minutes, its absorbance was measured at a wavelength of 570 nm.

### 2.9 Statistical analysis

In this study, statistical analyses were conducted using SPSS 23.0. Before analysis, all data were checked for normal distribution and homogeneity of variance, and Ln-transformed if necessary. Duncan’s multiple range test was used to determine differences among treatments, while an independent sample t-test was used to compare two Cd levels. Correlation analysis was performed using Origin Pro software (version 2.6.1) to investigate the relationship between growth morphology and Cd stress in *P. zhennan*. Differences were considered significant at p<0.05. Principal component analysis (PCA) was conducted using Canoco 5 software to compare the effects of adding nutrients on *P. zhennan* under Cd stress. To evaluate the impact of nitrogen, phosphorus, and mixed addition on the stress resistance Cd stress, mentioned growth and physiological indicators were measured and analyzed using treatment effect (Treatment effect = (T − CK)/(CK) *100) analysis. According to (Cisse et al., 2021), the fuzzy subordinate function analysis as described in their study, utilized the following calculation formula: U(Xi) = (Xi - Xmin)/(Xmax - Xmin). In this formula, U(Xi) represents the value of the subordinate function analysis for a specific parameter, Xi denotes the value of that parameter, Xmin indicates the minimum value of the parameter, and Xmax represents the maximum value of the parameter.

## 3 Results

### 3.1 Growth and biomass change

#### 3.1.1 Variations of nutrients levels in soil, roots, shoots and leaves

The addition of N significantly increased the soil NH_4_^+^-N and NO_3_^–^N content compared to the CK (**Table 1**). However, the N addition led to decreased uptake of K, Cu, Mn, and Zn by the root system while promoting the uptake of Fe and Ca. It was observed that in the presence of CdH stress, Cd-contaminated soil caused the release of more Cd^2+^, which facilitated Cd transfer to the root system. Cd may have entered the roots by replacing transporters for Cu, Fe, Ca, and Zn. This resulted in increased Cu, Fe, and Ca content in primary roots (**Supplementary Table S1**), with a significant increase in Fe. Secondary roots also exhibited elevated Cu, Fe, and Ca content, particularly in Fe and Ca (**Supplementary Table S2**). Tertiary roots showed increased Cu, Fe, Zn, and Ca content, with significant levels of Fe and Ca increase (**Supplementary Table S3**). Furthermore, Cd was predominantly distributed within the root system, entering the xylem. It was subsequently transported via water transpiration and accumulated in aboveground tissues, such as stems (**Supplementary Table S4**) and leaves (**Supplementary Table S5**). Cd promoted its transfer from the root system to stems by binding to Fe and Zn transporters. Under Cd stress, the increase in Fe and Ca content in leaves was significant due to the reduced transfer of Cd from stems to leaves. Additionally, CdHN treatment significantly increased total nitrogen (TN) content in primary, secondary, and tertiary roots compared to CdH. However, CdHN reduced TN content in the root system, particularly in tertiary roots, when compared to N treatment without Cd addition. Furthermore, CdHN significantly promoted the uptake of K and Fe in primary roots, as well as K, Fe, Mn, and Ca in secondary roots, along with Fe uptake in tertiary roots. However, CdHN led to a significant reduction in Fe content in leaves, consequently limiting the photosynthesis of *P. Zhennan* and hampering its growth and development.

**Table 1:** The change in soil NO_3_^–^-N and NH_4_^+^-N concentration among different treatments.

Treatment/soil	NO_3_^–^-N	NH_4_^+^-N
CK	2.67 ± 0.12b^*^	0.82 ± 0.12b^ns^
N	9.14 ± 1.31a^ns^	1.55 ± 0.15a^*^
P	3.24 ± 0.99b^ns^	1.36 ± 0.23a^ns^
NP	3.19 ± 0.18b^ns^	1.24 ± 0.08ab^ns^
CdH	3.29 ± 0.19B	0.91 ± 0.31A
CdHN	10.06 ± 1.33A	0.88 ± 0.16A
CdHP	2.70 ± 0.22B	0.90 ± 0.06A
CdHNP	4.58 ± 0.68B	1.19 ± 0.04A

Values are means ± standard error (n=3). Different letters indicate significant differences between the treatments (p<0.05) obtained via Duncan’s test. Lowercase letters indicate significant differences between different treatments under cadmium-free stress. Capital letters indicate significant differences between different treatments under cadmium stress. Asterisks denote statistically significant differences between the two Cd levels at p<0.05 according to independent-samples t-test (*: p<0.05; ns: p>0.05). CK: without N, P, and Cd; N: adding N, without P and Cd; P: adding P without N and Cd; NP: adding N and P without Cd; CdH: adding Cd without N and P; CdHN: adding Cd and N without P; CdHP: adding Cd and P without N; CdHNP: adding high Cd, N, and P.

Comparatively, P addition treatment significantly increased soil levels of K, Cu, Mn, Zn, and Ca. It notably reduced Zn content in primary roots, K, Cu, and Zn content in secondary roots, and K and Mn content in tertiary roots, while significantly increasing Fe content in roots. The addition of P facilitated the translocation of K, Mn, and Zn from roots to stems but significantly reduced Fe transfer. Overall, CdHP treatment significantly promoted root development, enhancing nutrient uptake and facilitating the translocation of K, Mn, and Zn from belowground to aboveground plant parts. However, leaf TN and Ca content in CdHP-treated plants were significantly lower than those in CdH-treated plants. The P addition under Cd stress alleviated the Cd stress primarily by enhancing root system growth and development, thus reducing Cd concentration in various plant organs.

In comparison to the CK, the addition of both NP increased the TN content in roots and stems. However, the TN content in primary and tertiary roots was significantly lower than that observed in the N treatment. NP addition led to a significant decrease in Zn content in primary roots, as well as reductions in K and Cu content in secondary roots, and K and Mn content in tertiary roots. Simultaneously, it significantly increased Ca and Fe content in secondary roots, along with a notable increase in Fe content in tertiary roots. This nutrient transfer resulted in a significant reduction in leaf K and Cu content, while Ca content was significantly increased compared to the CK. Under CdHNP treatment, TN levels significantly increased in primary, secondary, and tertiary roots, as well as in stems and leaves of *P. Zhennan*. Additionally, Ca content in secondary roots saw a significant increase, while Fe content in leaves decreased significantly. The Zn content in both roots and leaves displayed an upward trend. In contrast to CdHN treatment, CdHNP significantly inhibited the transfer of Cd from stems to leaves, effectively mitigating the toxic effects of Cd on leaves. These results suggest a certain degree of Cd stress mitigation with CdHNP treatment.

#### 3.1.2 Variations of plant growth

The effects of exogenous N and P addition on the morphology and biomass of *P. zhennan* were significant, as shown in **Supplementary Table S6**. The application of N in the absence of Cd stress significantly diminished leaf dry weight (LDW), stem dry weight (SDW), AB, and TB compared to CK. It also moderately reduced IPH and IGD, by 31.50% and 19.87% respectively when compared to CK. Both P and combined NP treatments, without Cd, positively influenced growth characteristics; P application particularly boosted LDW by 27%, while NP treatment notably increased IPH and IGD by 57.39% and 28.42% respectively, whereas at the expense of SDW, AB, and TB, which were reduced by 43%, 23%, and 22% respectively. The biomass of *P. zhennan*, excluding RDW, was substantially inhibited under CdH compared to CK. Under the same stress, the application of N (CdHN treatment) significantly reduced IPH, SDW, AB, and TB by 59.1%, 36%, 44%, and 49% respectively. Conversely, the P application (CdHP treatment) markedly improved IGD, LDW, SDW, RDW, AB, and TB by 66.20%, 75%, 72%, 48%, 73%, and 63% respectively when compared with CdH. Our findings demonstrated that Cd stress significantly hindered SDW and AB in comparison to Cd-free addition. In accordance, P addition was found to promote *P. zhennan* growth irrespective of Cd stress, whereas N addition exhibited an inverse effect.

#### 3.1.3 Variations of root order growth

In the absence of Cd stress, N treatment was found to significantly reduce the dry weight of the 1st, 2nd, and 3rd roots in comparison to the CK treatment. Conversely, P treatment without Cd stress notably increased the dry weight of the 1st and 2nd roots by 45.58% and 27% respectively (**Table 2**). Under Cd stress, the application of N (CdHN treatment) markedly decreased the dry weight of the 1st, 2nd, and 3rd roots by 55.45%, 58.31% and 55.62% respectively compared to the Cd alone treatment. The P application under Cd stress (CdHP treatment) resulted in a significant increase in the dry weight of the 2nd and 3rd roots, exerting a positive impact on root biomass.

**Table 2:** The change in first, second, and third root dry weight of *P. zhennan* among different treatments.

Treatment	1^st^ RDW (g)	2^nd^ RDW (g)	3^rd^ RDW (g)
CK	1.34 ± 0.21a^ns^	1.74 ± 0.30a^ns^	1.81 ± 0.60a^ns^
N	0.50 ± 0.12b^ns^	0.54 ± 0.11b^ns^	0.44 ± 0.07b^ns^
P	1.95 ± 0.44a^ns^	2.21 ± 0.40a^ns^	1.76 ± 0.15a^ns^
NP	1.55 ± 0.06a^ns^	1.58 ± 0.23a^ns^	1.28 ± 0.19ab^ns^
CDH	1.50 ± 0.17AB	1.35 ± 0.14B	0.98 ± 0.13B
CDHN	0.66 ± 0.12C	0.56 ± 0.03C	0.44 ± 0.02C
CDHP	1.73 ± 0.08A	2.08 ± 0.34A	1.59 ± 0.26A
CDHNP	1.31 ± 0.09B	1.01 ± 0.10BC	0.84 ± 0.15BC

^1st^ RDW; first root dry weight, ^2nd^ RDW; second root dry weight, ^3rd^ RDW; third root dry weight. CK: without N, P, and Cd; N: adding N, without P and Cd; P: adding P without N and Cd; NP: adding N and P without Cd; CdH: adding Cd without N and P; CdHN: adding Cd and N without P; CdHP: adding Cd and P without N; CdHNP: adding high Cd, N, and P. The data were mean ± standard error (n = 3). Different letters indicate significant differences between the treatments (p<0.05) obtained via Duncan’s test. Lowercase letters indicate significant differences between different treatments under cadmium-free stress. Capital letters indicate significant differences between different treatments under cadmium stress.

The influence of N addition on the growth of *P. zhennan* roots was significant, resulting in considerable inhibition irrespective of Cd content (**Supplementary Table S7**). The RL, ARD, RS, and RV significantly decreased in the presence of N and Cd stress (CdHN treatment). In contrast to the CdH treatment, the CdHP treatment notably enhanced root development, increasing RS and RV by 17.54% and 29.59%, respectively. The CdHNP treatment exhibited a reduction trend, significantly decreasing ARD by 22.54%. Notably, after CdHP treatment, ARD and RV values were significantly higher, when compared to P treatment alone. Furthermore, in comparison to NP treatment, RL, ARD, and RS values were significantly lower in CdHNP treatment.

#### 3.1.4 Treatment effect analysis

**Figure 1:**
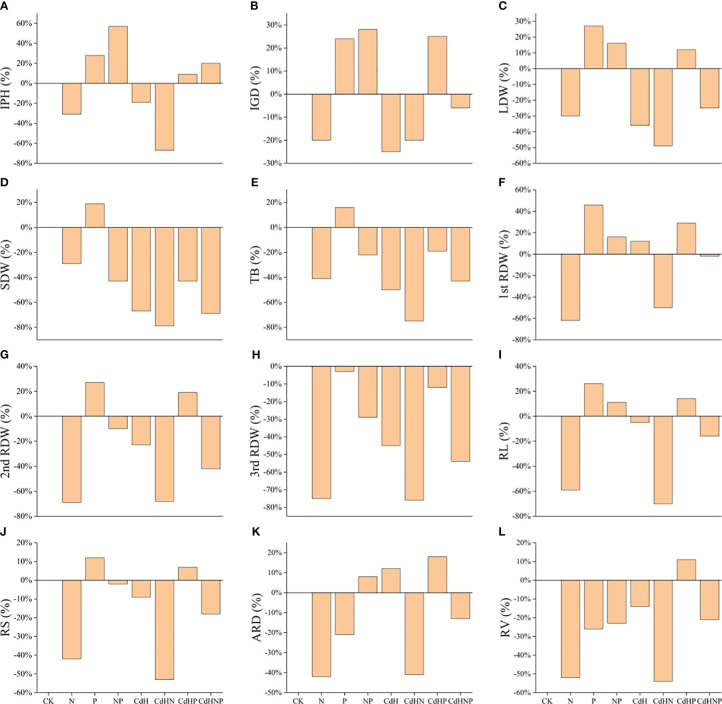
Treatment effect (TE) analysis among physiological, morphological, and biochemical indicators under N, P, and NP addition and Cd stress. **(A)** TE on IPH, **(B)** TE on IGD, **(C)** TE on ARD, **(D)** TE on AB, **(E)** TE on TB, **(F)** TE on RL, **(G)** TE on RV, **(H)** TE on LDW, **(I)** TE on SDW, **(J)** TE on 1^st^ RDW, **(K)** TE on 2^nd^ RDW, and **(L)** indicating TE on 3^rd^ RDW. Abbreviations include; IPH, plant height; IGD, ground diameter; LDW, leaf dry weight; SDW, stem dry weight; RDW, root dry weight; AB, the aboveground biomass; TB, total biomass; 1^st^ RDW, 1^st^ root dry weight, 2^nd^ RDW, 2^nd^ root dry weight and 3^rd^ RDW, 3^rd^ root dry weight; RL, root length; ARD, average root diameter; RS, root surface; RV, root volume; Pn, net photosynthesis rate; Gs, stomatal conductance; Ci, intercellular CO_2_ concentration; Tr, transpiration; Chla, chlorophyll a; Chlb, chlorophyll b; TChl, total chlorophyll; Caro, carotenoid; O_2_^·−^, superoxide radicals; MDA, malondialdehyde; RC (%), relative conductivity; SP, soluble protein; Pro, proline; GSH, glutathione; POD, peroxidase; SOD, superoxide dismutase; CAT, catalase; APX, ascorbic acid peroxidase.

We analyzed the treatment effect of N, P, and mixed supplementation on the resistance of *P. zhennan* to Cd stress, with results summarized in **Figure 1**. The P supplementation enhanced various growth indicators including: IPH, IGD, AB, TB, RL, RV, LDW, 1st RDW, and 2nd RDW relative to the CK. Conversely, the N supplementation, regardless of Cd stress, generally hindered growth potential due to nitrogen toxicity. However, a combined N and P supplementation ameliorated several traits including; IPH, IGD, ARD, RL, and 1st RDW, indicating a potential synergistic response to nitrogen toxicity.

Furthermore, CdHP treatment positively influenced IPH, IGD, ARD, RL, RV, LDW, 1st RDW, and 2nd RDW compared to CdH treatment. Moreover, the CdHNP treatment, when compared to CdHN, improved all measured traits (IPH, IGD, ARD, AB, TB, RL, RV, LDW, SDW, 1st RDW, 2nd RDW, 3rd RDW), suggesting a synergistic effect in coping with nitrogen toxicity.

### 3.2 Gas exchange and the chlorophyll concentration

**Table 3** reveals the influence of Cd, N, and P on the photosynthetic parameters of *P. zhennan* seedlings. The N treatment, in the absence of Cd, significantly decreased Chla by 51.74%, Chlb by 62.28%, and TChl by 53.20%. However, it only slightly influenced the Pn, Gs, Ci, Tr, and Caro by 7.6%, 7.9%, 2.0%, 3.6%, and 37.8% respectively. The absence of Cd also saw the P and NP treatments significantly enhance the Tr by 58.02% (p<0.05) compared to the CK treatment. In contrast, Cd stress significantly reduced the photosynthetic Pn by 21.04%, accompanied by decreases in Chl a, b, and TChl levels when compared to the CK treatment. Finally, the CdHN treatment resulted in decreased levels of Pn, Gs, Ci, and Tr while increasing the levels of Chla, Chlb, TChl, and Caro compared to CdH.

**Table 3:** Gas exchange and the chlorophyll concentration of *P. zhennan* among different treatments.

Treatment	Pn (μmol· m^-2^·s^-1^)	Gs (mol· m^-2^·s^-1^)	Ci (μmol· mol^-1^)	Tr (mol· m^-2^·s^-1^)	Chla (mg· g^-1^·FW)	Chlb (mg· g^-1^·FW)	Total Chl (mg· g^-1^·FW)	Caro (mg· g^-1^·FW)
CK	8.74± 0.57a^*^	0.08± 0.01a^ns^	242.11± 10.75a^ns^	1.58± 0.13c^ns^	1.72± 0.25a^ns^	0.28± 0.04a^ns^	2.00± 0.29a^ns^	0.39± 0.07ab^ns^
N	8.08± 0.24a^ns^	0.08 ± 0.01a^ns^	237.33± 26.10a^ns^	1.64± 0.09bc^ns^	0.83± 0.12b^*^	0.10± 0.02b^**^	0.94± 0.15b^*^	0.24± 0.02b^*^
P	9.05± 0.81a^ns^	0.10 ± 0.01a^ns^	252.60± 38.97a^ns^	2.00± 0.07b^*^	1.79± 0.28a^ns^	0.26± 0.03a^*^	2.05± 0.31a^ns^	0.46± 0.08ab^ns^
NP	9.53± 0.44a^*^	0.09± 0.01a^ns^	223.46± 3.90a^ns^	2.50± 0.14a^*^	2.30± 0.33a^ns^	0.34± 0.06a^ns^	2.65± 0.39a^ns^	0.55± 0.08a^ns^
CdH	6.90± 0.26AB	0.06± 0.00AB	247.84± 8.26AB	1.57± 0.13B	1.48± ± 0.19B	0.25± 0.03B	1.73± 0.22A	0.39± 0.03A
CdHN	6.26± 0.73B	0.05± 0.01B	222.58± 4.76B	1.37± 0.20B	1.77± 0.20AB	0.28± 0.03AB	2.05± 0.23A	0.44± 0.04A
CdHP	7.11± 0.39AB	0.07± 0.01AB	261.96 9.49A	1.61± 0.11AB	1.91± 0.07AB	0.37± 0.01A	2.28± 0.08A	0.46± 0.01A
CdHNP	7.90± 0.10A	0.07± 0.01A	245.98± 8.38AB	2.05± 0.07A	2.05± 0.14A	0.33± 0.04AB	2.38± 0.18A	0.50± 0.04A

Pn, net photosynthesis rate; Gs, stomatal conductance; Ci, intercellular CO2 concentration; Tr, transpiration; Chla, chlorophyll a; Chlb, chlorophyll b; TChl, total chlorophyll; Caro, carotenoid CK: without N, P, and Cd; N: adding N, without P and Cd; P: adding P without N and Cd; NP: adding N and P without Cd; CdH: adding Cd without N and P; CdHN: adding Cd and N without P; CdHP: adding Cd and P without N; CdHNP: adding high Cd, N, and P. The data were mean ± standard error (n = 3). Different letters indicate significant differences between the treatments (p<0.05) obtained via Duncan’s test. Lowercase letters indicate significant differences between different treatments under cadmium-free stress. Capital letters indicate significant differences between different treatments under cadmium stress. Asterisks denote statistically significant differences between with and without Cd under the same treatments at p<0.05 according to independent-samples t-test (*: p<0.05; **: p<0.01; ns: p>0.05).

### 3.3 Physiological and biochemical changes

#### 3.3.1 Variations of oxidative stress markers

**Figure 2:**
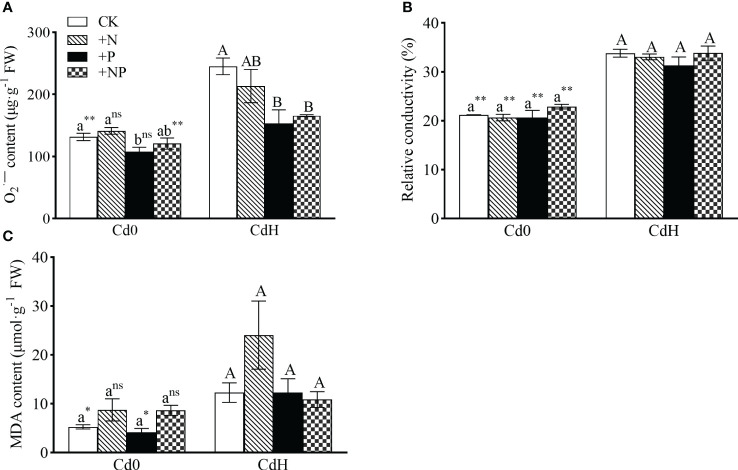
The effects of adding N, P, and Cd on O2^·−^ **(A)**, relative conductivity **(B)**, and MDA **(C)** of *P. zhennan* among different treatments. Abbreviations include O2^·−^; superoxide radicals, MDA; malondialdehyde. Values are means ± standard error (n=3). Different letters on the bars indicate significant differences between the treatments (p<0.05) obtained via Duncan’s test. Lowercase letters indicate significant differences between different treatments under cadmium-free stress. Capital letters indicate significant differences between different treatments under cadmium stress. Asterisks denote statistically significant differences between the two Cd levels at p<0.05 according to independent-samples t-test (*: p<0.05; **: p<0.01; ns: p>0.05). CK: without N, P, and Cd; N: adding N, without P and Cd; P: adding P without N and Cd; NP: adding N and P without Cd; CdH: adding Cd without N and P; CdHN: adding Cd and N without P; CdHP: adding Cd and P without N; CdHNP: adding high Cd, N, and P.

The study demonstrated distinct alterations in O_2_^·−^ and MDA levels influenced by N, P, and Cd treatment conditions. However, P supplementation without Cd significantly mitigated O_2_^·−^ level. Cd stress exhibited a notable increase (p ≤ 0.05) in O_2_^·−^, relative conductivity, and MDA by 86.45%, 59.45%, and 132.62% respectively. Remarkably, both CdHP and CdHNP treatments mitigated O_2_^·−^, showing a significant inter-treatment difference (**Figure 2**).

#### 3.3.2 Variations of osmotic regulating substances

**Figure 3:**
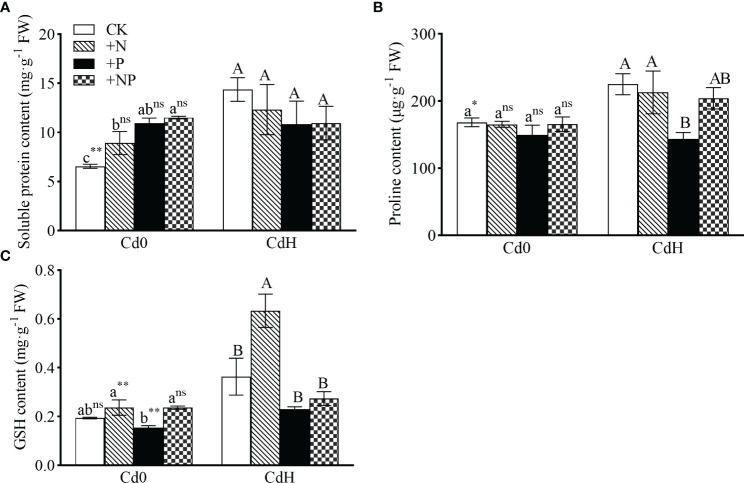
The effects of adding N, P, and Cd on soluble protein **(A)**, proline **(B)**, and GSH content **(C)** of *P. zhennan* among different treatments. Abbreviations include GSH; glutathione synthetase. The data were mean ± standard error (n=3). Lowercase letters indicate significant differences between different treatments under cadmium-free stress. Capital letters indicate significant differences between different treatments under cadmium stress. Asterisks denote statistically significant differences between the two Cd levels at p<0.05 according to independent-samples t-test (*: p<0.05; **: p<0.01; ns: p>0.05). CK: without N, P, and Cd; N: adding N, without P and Cd; P: adding P without N and Cd; NP: adding N and P without Cd; CdH: adding Cd without N and P; CdHN: adding Cd and N without P; CdHP: adding Cd and P without N; CdHNP: adding high Cd, N, and P.

The findings show substantial alterations in soluble protein, proline, and GSH levels subjected to different N, P, and Cd treatment setups. In the absence of Cd, the addition of N, P, and NP significantly enhanced soluble protein content by 36.20%, 67.36%, and 75.86% respectively compared to CK (**Figure 3**). Cd stress led to a statistically significant rise (p ≤ 0.05) in soluble protein (119.6%) and proline (33.75%) levels, coupled with a 22.52% increment in GSH compared to CK. Compared to CdH treatment, CdHN resulted in a noteworthy enhancement in GSH content by 74.32% (p ≤ 0.05, **Figure 2C**). A significant reduction (p ≤ 0.05) in proline content by 36.18% was particularly noticeable in the CdHP treatment compared to CdH (**Figure 3B**).

#### 3.3.3 Variations of enzymes activities

**Figure 4:**
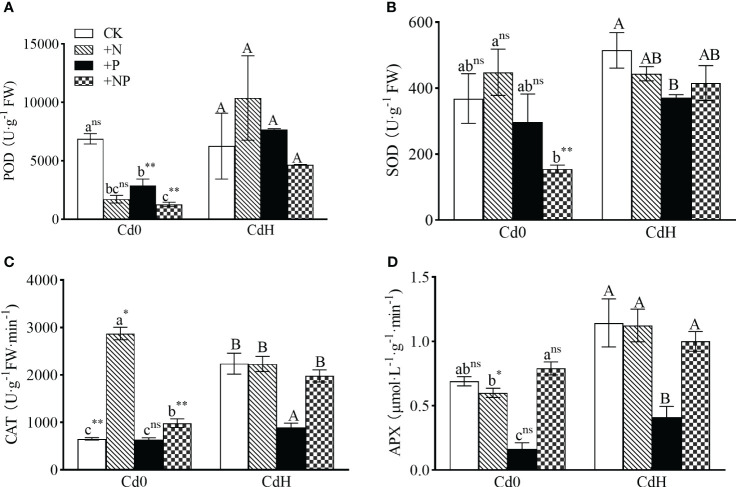
The effects of adding N, P, and Cd on POD **(A)**, SOD **(B)**, CAT **(C)**, and APX **(D)** activities of *P. zhennan* among different treatments. The data were mean ± standard error (n=3). Lowercase letters indicate significant differences between different treatments under cadmium-free stress. Capital letters indicate significant differences between different treatments under cadmium stress. Asterisks denote statistically significant differences between the two Cd levels at p<0.05 according to independent-samples t-test (*: p<0.05; **: p<0.01; ns: p>0.05). CK: without N, P, and Cd; N: adding N, without P and Cd; P: adding P without N and Cd; NP: adding N and P without Cd; CdH: adding Cd without N and P; CdHN: adding Cd and N without P; CdHP: adding Cd and P without N; CdHNP: adding high Cd, N, and P.

In the absence of Cd, the supplementation of N and NP substantially elevated CAT activity by 75.02% and 441% respectively, while concurrently reducing POD activity, as compared to the CK (p<0.05, **Figure 4**). The P treatment without Cd, however, notably reduced POD (57.87%) and APX (76.33%) activity (p<0.05). Exposure to Cd stress triggered a significant surge in CAT (242.76%), SOD, and APX activity (**Figures 4B–D**) compared to CK. Comparatively, CdHP treatment lowered SOD (27.96%), CAT (60.06%), and APX (64.14%) activity compared to CdH, while significantly enhancing POD activity by 164.82% when compared to cadmium-free P treatment (p<0.05). Nonetheless, CdHN treatment notably decreased CAT activity by 22.31% but significantly boosted APX activity by 87.22% compared to cadmium-free N treatments. In addition, CdHN treatment decreased the SOD activity significantly compared to CdH. Further, CdHNP treatment considerably increased POD, SOD, and APX activity by 266.36%, 168.44%, and 26.58% respectively when compared to cadmium-free NP treatment.

### 3.4 Variations of Cd levels

**Figure 5:**
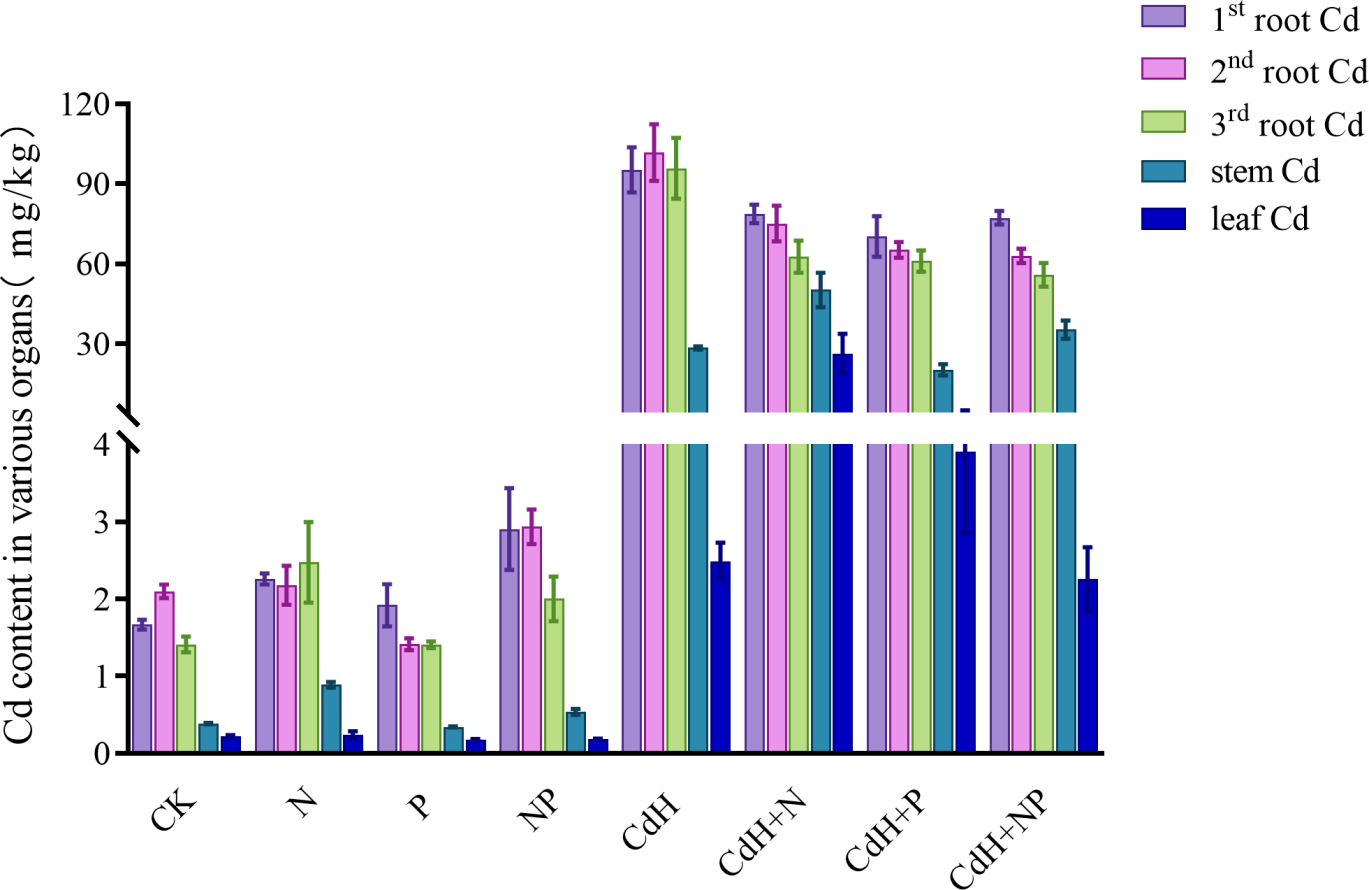
Cd levels in different organs of *P. zhennan* among different treatments. CK: without N, P, and Cd; N: adding N, without P and Cd; P: adding P without N and Cd; NP: adding N and P without Cd; CdH: adding Cd without N and P; CdHN: adding Cd and N without P; CdHP: adding Cd and P without N; CdHNP: adding high Cd, N, and P.

The Cd levels of *P. zhennan* in various organs under N, P, and Cd treatments are depicted in **Supplementary Table S8**, **Figure 5** calculated at the end of the experiment. The values of the Cd contents were in the following order: 2nd roots > 1st roots > 3rd roots > stems > leaves, indicating that under Cd-free stress, the 2nd roots had the highest Cd content and the leaves had the lowest. When N was added without Cd, Cd was more readily absorbed and accumulated in many organs, and the levels of the stem (61.96%) and third roots (57.44%) were significantly different. Similar to this, the addition of NP without Cd treatment markedly enhanced Cd accumulation in the stem, first root, and second root by 22.17%, 100%, and 24.86% respectively. However, P addition without Cd stress significantly reduced Cd accumulation in the second root.

Upon CdH treatment, the Cd content was the highest in the roots and the lowest in the leaves. Interestingly, both CdHN and CdHP treatments augmented Cd accumulation in the stem and leaf, while slightly decreasing it in the roots. However, CdHNP treatment substantially mitigated Cd accumulation in leaves compared to CdH.

### 3.5 Correlation of osmoregulatory substances, antioxidant enzymes, root architecture, and photosynthetic activities

**Figure 6:**
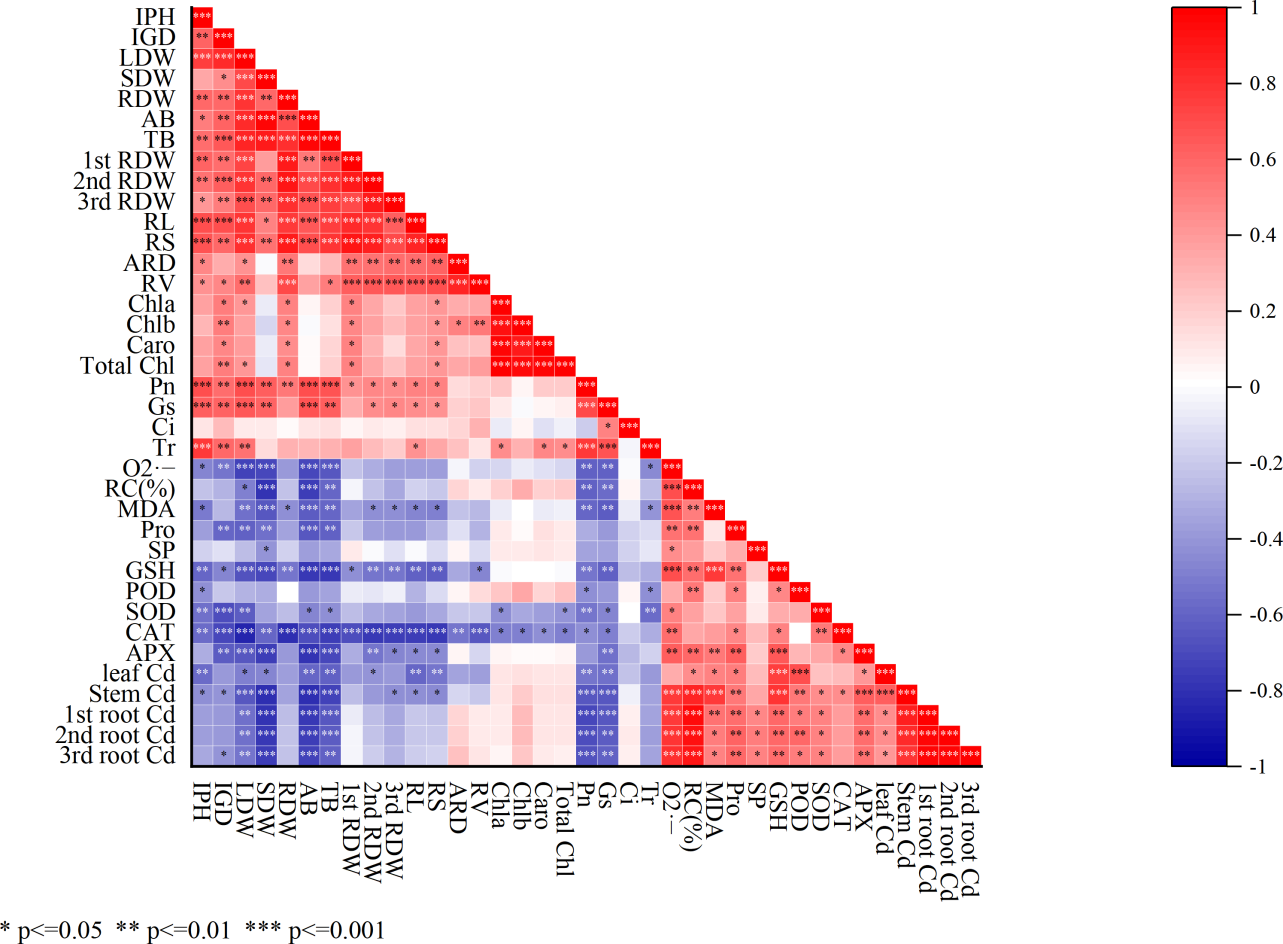
Correlation analysis among mentioned parameters. Calculated as *p<0.05, **p<0.01, and ***p<0.001. IPH, plant height; IGD, ground diameter; LDW, leaf dry weight; SDW, stem dry weight; RDW, root dry weight; AB, the aboveground biomass; TB, total biomass; 1^st^ RDW, 1^st^ root dry weight, 2^nd^ RDW, 2^nd^ root dry weight and 3^rd^ RDW, 3^rd^ root dry weight; RL, root length; ARD, average root diameter; RS, root surface; RV, root volume; Pn, net photosynthesis rate; Gs, stomatal conductance; Ci, intercellular CO_2_ concentration; Tr, transpiration; Chla, chlorophyll a; Chlb, chlorophyll b; TChl, total chlorophyll; Caro, carotenoid; O_2_^·−^, superoxide radicals; MDA, malondialdehyde; RC (%), relative conductivity; SP, soluble protein; Pro, proline; GSH, glutathione; POD, peroxidase; SOD, superoxide dismutase; CAT, catalase; APX, ascorbic acid peroxidase.

The correlation analysis depicted that, the osmoregulatory substances including Pro, SP, and GSH had a significant positive correlation (*p<0.05, **p<0.01, ***p<0.001) with antioxidant enzymes activities including POD, SOD, CAT, and APX (**Figure 6**). On the other hand, a significant negative correlation was shown among antioxidant enzymes (POD, SOD, CAT, and APX) with SDW, LDW, RDW, AB, and TB. However, the root architecture RL and RV showed a negative correlation with the 1^st^ RDW, 2^nd^ RDW, and 3^rd^ RDW and photosynthetic activities including stomatal conductance, transpiration, and intracellular CO_2_.

### 3.6 PCA of growth, fresh weight, photosynthesis, physiology, and root biomass factors of different root orders

**Figure 7:**
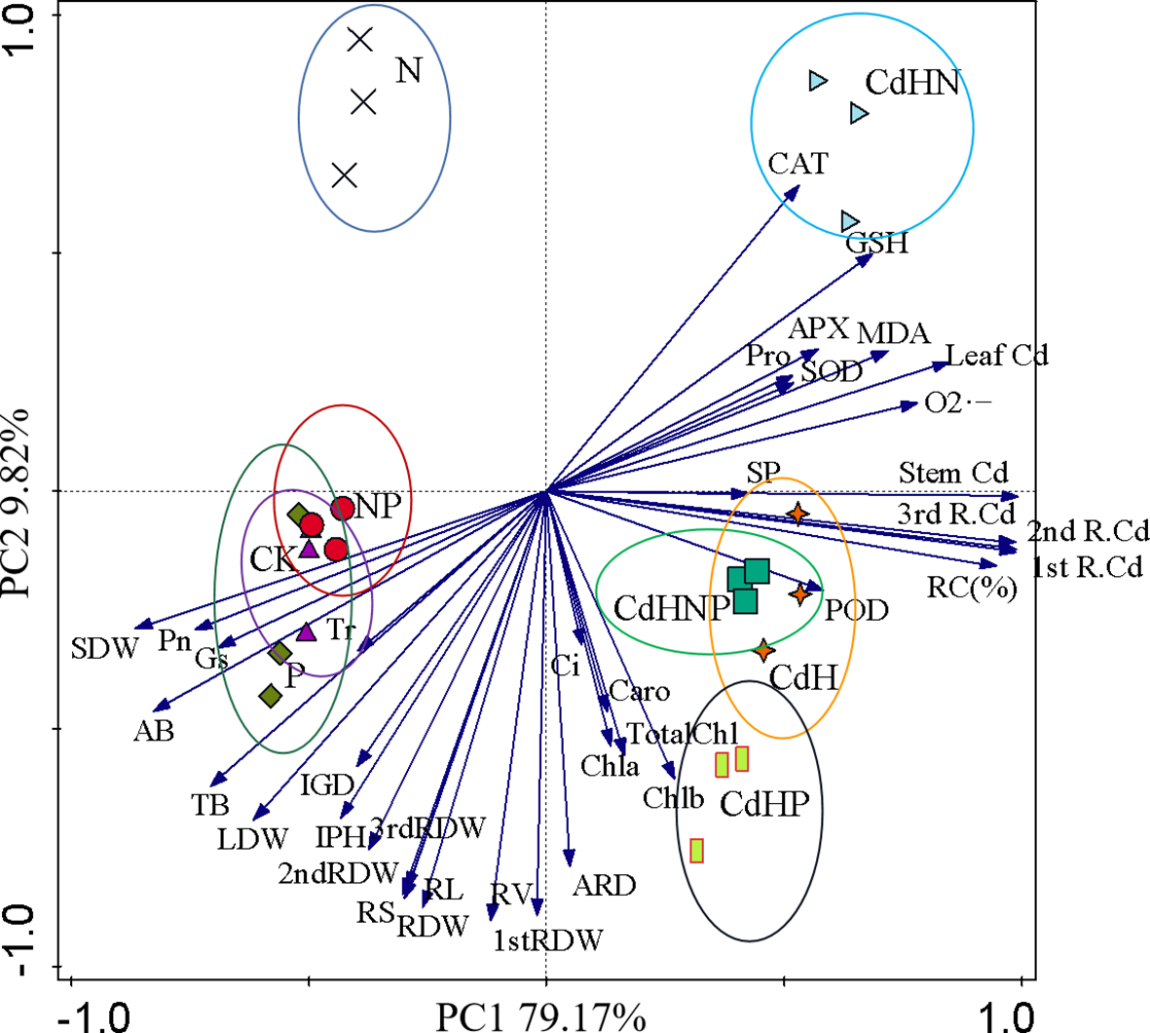
Principal component analysis (PCA) based on the growth, fresh weight, photosynthesis, physiology, and root biomass factors of different root orders of *P. zhennan* seedlings under cadmium-free and added cadmium stress. (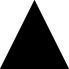) Indicating the CK treatment group; (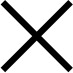) indicating the N treatment group; (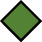) indicating the P treatment group; (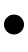) indicating the NP treatment group; (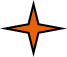) indicates the CdH treatment group (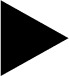) indicating the CdHN treatment group (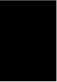) indicating CdHP treatment group; (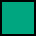) indicating CdHNP treatment group.

PCA based on the growth, dry weight, photosynthesis, physiology, and root biomass factors of different root orders of *P. zhennan* seedlings under cadmium-free and added cadmium stress was conducted (**Figure 7**). The results of PCA convincingly demonstrated the impact of mixed addition of N, P, and NP on *P. zhennan* seedling growth and physiology under both Cd-free and added Cd stress. The cumulative contribution of PC1 and PC2 was 88.99%. Pn indicated a significantly negative correlation with CAT, GSH, POD, LDW, 2nd RDW, RV, and a significant positive correlation with SDW, AB, TB, LDW, and RV. Furthermore, no noticeable correlation across each indicator under CdH and CdHN treatments was found.

### 3.7 Fuzzy differential subordination analysis among physiological, morphological, and biochemical indicators

To evaluate the impact of nitrogen, phosphorus, and mixed addition on the stress resistance Cd stress, various growth and physiological indicators were measured and analyzed using the fuzzy subordinate function. The results are summarized in **Supplementary Table S9**, where higher mean values of the comprehensive evaluation indicate better plant growth and greater resistance to abiotic stress. The comprehensive evaluation values for each treatment under Cd-free stress were found to be in the order of P > NP > CK > N, indicating that phosphorus had the most positive effect on promoting the growth and development of *P. zhennan*. Under Cd stress, the order of comprehensive evaluation values was found to be CdHP > CdHNP > CdH > CdHN. This suggests that CdHP and CdHNP treatments can alleviate the negative effects of Cd stress, with CdHP having the most significant effect.

## 4 Discussion

### 4.1 Preferences for fertilizer and variations in the growth of *P. zhennan*

The preference of plants for different forms of N for optimal growth is an evolved characteristic, with some favoring NH_4_^+^-N and others NO_3_^–^N (Guo et al., 2022). Yet, an excess of both NH_4_^+^ and NO_3_^–^ can pose harmful consequences. For instance, a 75:25 ratio of NO_3_^–^-N and NH_4_^+^-N is the most beneficial, while any surfeit of NH_4_^+^-N impeded growth (Huang et al., 2016). Our investigation into *P. zhennan* seedlings revealed higher NH_4_^+^-N content in leaves (**Supplementary Figure S1**) when N and NP were applied with the NO_3_^–^-N content correspondingly lower compared to the control group. These results suggest that *P. zhennan* seedlings demonstrate a preference for NH_4_^+^-N and a diminished ability to utilize NO_3_^–^-N. Indeed, high NO_3_^–^-N concentrations may have toxic effects that inhibit growth.

The N supplementation has been shown to enhance plant growth and stress resistance, including to Cd, but excessive N lead to issues such as plant lodging and root strength reduction (Haynes and Goh, 1978; Mizuta et al., 2020; Aye and Masih, 2023; Raza et al., 2023). It has impacted plant root systems directly and indirectly by altering soil N availability (Galloway et al., 2004), which is pivotal for root dynamics (Vogt et al., 1995). An increase in soil N has been linked to decreased fine root biomass (Galloway et al., 2004; Hendricks et al., 2006). In our study, we observed that the N addition inhibited *P. zhennan* growth and induced negative responses under Cd stress (**Figure 2**), contradicting some previous findings and calling for further investigations into the underlying mechanisms. Excess N application decreased leaf LDW, stem SDW, AB, and TB significantly and reduced IPH and IGD moderately, pointing towards potential nutrient imbalance (Galloway et al., 2004; Atkinson and Urwin, 2012; Sinclair and Rufty, 2012). Under Cd stress, N application (CdHN treatment) further decreased PH, SDW, AB, and TB, in line with prior research suggesting Cd stress disrupts nitrogen metabolism in plants (DalCorso et al., 2010). This differential response of growth parameters to Cd stress and N application indicates a possible unknown stress-response mechanism in plants which needs further exploration. The possibility of an unknown self-defence mechanism triggered by Cd stress and N application offers an intriguing avenue for future research. The observed negative response could be due to N toxicity (Goyal and Huffaker, 1984), though the detoxification response and underlying mechanism are yet to be elucidated. Our findings have significant ecological implications, suggesting a complex interplay of nutrients and stressors that may redefine our understanding of plant responses to environmental stressors.

Studies show that Cd inhibits plant growth even in the presence of N fertilizers (Yao et al., 2012). Confirming this, our research found that the groups treated with high concentrations of Cd (CdH, CdHN, and CdHNP) exhibited the lowest plant heights. Yet, P treatment under Cd stress has been reported to increase plant dry matter mass (Chia et al., 2015; Gong et al., 2019; Maqbool et al., 2022; Wang et al., 2022), possibly due to P’s ability to form insoluble phosphates with Cd, thus limiting its toxicity. Intriguingly, N application under Cd stress was found to enhance Cd absorption in leaves, while P application concurrently with fertilizers decreased it. This points to a synergistic interaction between N and P applications, as well as N and limited Cd, in mitigating the toxic effects of N application.

### 4.2 The N and P addition regulates Cd compartmentalization in tree organs

Plants have been observed to naturally compartmentalize toxic elements like Cd, predominantly in roots to reduce their translocation to aerial parts (Shi et al., 2019). Our study concurs with these findings showing Cd accumulation in 2nd roots > 3rd roots > 1st roots > stems > leaves under CdH conditions. The N addition, in the absence of Cd, led to a rise in Cd accumulation in several organs, possibly due to N synergy in Cd uptake and translocation (Tie et al., 2019; Jia et al., 2020). This effect might also be associated with N’s role in enhancing root hair formation and surface area available for nutrient uptake including Cd (Zhou et al., 2020). Meanwhile, the addition of NP without Cd treatment notably increased Cd accumulation in the stem, 1st root, and 2nd root. This increase may be attributed to the enhanced nutrient uptake and translocation capacity under NP treatment (Dai et al., 2018), and potentially the promotion of root exudates aiding Cd uptake (Jia et al., 2020). In contrast, the addition of P alone significantly reduced Cd accumulation, possibly due to the competitive inhibition of Cd uptake by P and the formation of less absorbable Cd-P complexes (Tariq et al., 2017). Moreover, especially under the addition of NP, the concentration of Cd in roots of different root sequences under CdH was reduced, thereby reducing its impact on the normal function of roots and improving the ability of *P. Zhennan* to resist Cd pollution (**Figure 5**).

### 4.3 The N and P coordinately promote defense against Cd stress

Our investigation reveals that both N and Cd act as stressors, adversely impacting *P. zhennan*. Notably, N supplementation appears to intensify the Cd stress effect. When combined, the detriments to *P. zhennan* exceed those imparted by either Cd or N alone, yet the observed inhibitory effect falls short of a simple additive expectation. Intriguingly, the co-supplementation of N and P seems to mitigate the stress effects imposed by cadmium and nitrogen, underscoring the potential benefits of balanced nutrient strategies for stress resistance.

This study confirms that elevated Cd levels in both CK and CdH treatments reduce chlorophyll content adversely affecting photosynthetic activity (Chen et al., 2020; Zhou et al., 2020). However, we found that N and P supplementation can counteract these deleterious effects, bolstering chlorophyll and carotenoid content, and thereby enhancing photosynthetic and transpiration processes. It has been demonstrated that phosphorus application can amplify stomatal conductance and water use efficiency (Liu and Guo, 2013). Our study aligns with these findings, with P and combined NP fertilizers enhancing stomatal conductance in *P. zhennan* leaves. However, contrary to expectations our results showed a decrease in water use efficiency. In the presence of Cd, no significant difference was observed in transpiration rate between CK and CdH treatments suggesting that fertilization ameliorates transpiration rate, while Cd stress impairs photosynthetic rate and water use efficiency. Our study concurs with (El Rasafi et al., 2022), that Cd stress can lower the net photosynthetic rate and stomatal conductance of leaves. The data underscore the assertion that Cd stress predominantly hampers plant photosynthesis and that non-stomatal factors primarily constrain leaf transpiration rates.

The detrimental accumulation of ROS under stress, leading to potential plant death, is well-documented (Ayala et al., 2014). Our results extend this understanding by demonstrating that N supplementation can both exacerbate and ameliorate ROS accumulation, depending on the presence of Cd. We further observed that P and NP treatments consistently suppress ROS generation, which together with the observed reduction in MDA content (El Rasafi et al., 2022), suggests effective stress mitigation.

While the role of plant antioxidants in mitigating Cd stress is well recognized (Yang et al., 2020), inconsistency prevails across studies examining the response of the antioxidant system to stress. Certain findings substantiate that the optimal P ratio enhances plant biomass, curtails oxidative stress, and augments antioxidant enzyme activity (Jiang et al., 2020; Maqbool et al., 2022). Our research echoes these observations noting an initial increase and subsequent decrease in POD activity with escalating Cd concentration in celery seedlings. Conversely, the SOD, CAT, and POD activities in *Dendrobium officinale* show an upward trend with rising Cd concentration, an outcome in alignment with typical plant stress responses (Jiang et al., 2020). Intriguingly, the CAT and POD activities in pepper seedlings initially recede and then advance, while the SOD activity consistently declines with an upward Cd content trajectory (Al-Khayri et al., 2022). A similar downward trend in SOD and POD activities is observed in *Salix viminalis* with increasing Cd content. However, the application of NP enhances the activities of all three antioxidant enzymes, deviating from our observations. The Cd exposure reportedly diminishes SOD activity and elevates MDA content in soybeans, while P application inversely impacts these parameters (Dixit et al., 2001). In contrast, our findings demonstrate an upsurge in these enzyme activities upon NP addition signifying an escalation in oxidative stress due to Cd exposure. The elevated MDA levels further underscore this oxidative stress manifestation, indicative of lipid peroxidation (Jia et al., 2020; Maqbool et al., 2022). However, the amalgamated NP application mitigates Cd stress, increases plant biomass, and substantially reduces MDA content.

Finally, we investigated the impacts of N, P, and NP supplementation on soluble protein, proline, and GSH levels under Cd stress. The observed increase in protein levels with N, P, and NP treatment, while proline and GSH levels remained relatively stable, aligns with existing literature (Hasanuzzaman et al., 2020). Interestingly, under Cd stress, these stress-responsive molecules were found to increase, indicating activation of plant defense mechanisms (Jia et al., 2020). However, Cd and P supplementation resulted in lower stress-responsive molecule levels, hinting at potential uptake competition (Grossiord et al., 2020; Jang et al., 2020). This point certainly merits further investigation.

### 4.4 Root morphology and exudation of metabolites

Plants employ adaptive strategies such as root morphology modulation and metabolite exudation to augment tolerance to heavy metal stress (Liu et al., 2020). This research demonstrates that the *P. zhennan* root system encompassing RL, ARD, RS, and RV, exhibits significant growth under P treatment amidst Cd stress, indicating the potential of P to bolster root growth under heavy metal stress. This growth is particularly noticeable in the 1st root, which is crucial for nutrient absorption and supporting plant growth (Kong et al., 2010; Long et al., 2013). Interestingly, the 2nd and 3rd roots are associated with Cd enrichment, thereby enhancing stress tolerance via MDA level reduction, chlorophyll content increase, and promotion of transpiration and photosynthesis. Conversely, the N application appears to dampen these root growth parameters, intensifying stress and modifying root morphological characteristics. The root is the primary organ for Cd accumulation in plants (Dai et al., 2018). Generally, exposure to both N and P causes a decrease in the root-to-shoot ratio, reflecting higher biomass allocation to above-ground structures due to N and P stimulatory effect on shoot growth, coupled with Cd inhibitory effect on root growth. Nevertheless, under Cd stress, our study shows a significant increase in the root-to-shoot biomass ratio of *P. zhennan* seedlings (p<0.05), which tends to decline under N and P application. However, this response can fluctuate based on the plant species (Liu et al., 2018), Cd concentration, nutrient levels, and environmental conditions.

Our findings demonstrate the complex interplay between N, P, and Cd on root growth. Consistent with previous studies, excessive N supply decreased root dry weight, while P treatment enhanced root growth (Tariq et al., 2017; Tariq et al., 2019). Cd stress combined with high N levels had a synergistic negative effect on root growth (Li et al., 2017), whereas phosphorus alleviated Cd toxicity by activating Cd uptake and stimulating detoxification mechanisms (Jia et al., 2020). However, the combined N and P treatment mitigates Cd’s impact on root growth, suggesting contrasting or interactive effects of N and P under Cd stress. Further research is needed to elucidate the molecular and physiological mechanisms underlying these interactions and optimize nutrient management strategies for plants under heavy metal stress.

## 5 Conclusions and recommendations

Our investigation reveals that the exogenous application of P and mixed NP can confer a protective effect on plants under Cd stress, a finding with significant implications for mitigating heavy metal toxicity in plants and effectively utilizing Cd-contaminated lands for tree plantations. The protective effect is primarily associated with enhanced root development, which in turn leads to a balanced nutrients absorption and notable decrease in MDA levels by 21.48%. Concurrently, the chlorophyll content exhibited a substantial increase of 53.20%, accompanied by a transpiration promotion of 58.02% and photosynthesis augmentation of 7.6%. The augmentations in these vital physiological processes indicate an overall improvement in plant health under Cd stress. Further, the combined application of N and P was found to stimulate the activities of antioxidant enzymes indicating an increased capacity for oxidative stress defense. This finding offers valuable insight into the role of nutrient supplementation in enhancing the tolerance of *P. zhennan* to Cd. Nevertheless, caution must be exercised as excessive application of N and P might disrupt the nutrient balance negatively impacting the heavy metal detoxification capabilities of the plants. Furthermore, the specific form of N and P applied can significantly influence their effectiveness in promoting Cd detoxification, regulatory mechanisms for Cd uptake, translocation, accumulation span root uptake, and intra-plant transport. The availability of N and P can modulate the expression of transporters and proteins instrumental in Cd uptake and accumulation. Understanding these complex mechanisms can aid researchers in formulating strategies to reduce Cd accumulation. This might involve choosing plant varieties with lower Cd absorption or refining soil management techniques. Therefore, this study emphasizes the fine line between nutrient addition and metal detoxification, a balance essential for sustainable plant health and the efficient use of Cd-contaminated lands amidst growing environmental concerns.

## Data availability statement

The original contributions presented in the study are included in the article/**Supplementary Material**. Further inquiries can be directed to the corresponding authors.

## Author contributions

JZ: Conceptualization, Formal analysis, Investigation, Methodology, Software, Writing – original draft, Writing – review & editing. NS: Data curation, Formal analysis, Validation, Writing – original draft, Writing – review & editing. KL: Data curation, Investigation, Methodology, Software, Writing – review & editing. NM: Data curation, Formal analysis, Validation, Writing – review & editing. XW: Data curation, Formal analysis, Investigation, Writing – review & editing. XS: Formal analysis, Supervision, Writing – review & editing. LZ: Formal analysis, Supervision, Writing – review & editing. KP: Conceptualization, Funding acquisition, Supervision, Validation, Writing – review & editing.
