# Supplementary material for: Boosting cadmium tolerance in Phoebe zhennan: the synergistic effects of exogenous nitrogen and phosphorus treatments promoting antioxidant defense and root development
Source: Front Plant Sci. 2024 Feb 1;15:1340287. doi: 10.3389/fpls.2024.1340287 (PMC10867629; doi:10.3389/fpls.2024.1340287)
Supplement: Supplementary file 1 [file DataSheet_1.docx]

Supplementary Material

**Table S1. The change in total nitrogen, total carbon and various important nutrients in the first order roots of *Phoebe zhennan* among different treatments**.

| **Treatment/1^st^ root** | **TN (g/kg)** | **TC (g/kg)** | **K (g/kg)** | **Cu (mg/kg)** | **Fe (mg/kg)** | **Mn (mg/kg)** | **Zn (mg/kg)** | **Ca (g/kg)** |
| --- | --- | --- | --- | --- | --- | --- | --- | --- |
| CK | 19.70±0.80bc^ns^ | 473.15±4.87a^ns^ | 1.38±0.14a^*^ | 47.03±2.95a^ns^ | 1816.39±250.15b^ns^ | 38.39±6.05a^*^ | 60.22±8.78a^*^ | 6.37±0.24a^ns^ |
| N | 29.49±1.39a^ns^ | 476.62±2.00a^*^ | 1.25±0.09a^ns^ | 32.73±4.06a^ns^ | 2332.19±311.94ab^**^ | 23.05±4.24b^ns^ | 29.87±3.24b^ns^ | 7.80±0.09a^ns^ |
| P | 18.09±1.10c^ns^ | 482.72±2.30a^*^ | 1.29±0.11a^ns^ | 50.16±14.49a^ns^ | 2930.66±299.09a^ns^ | 34.10±2.18ab^*^ | 31.65±4.62b^*^ | 7.49±0.88a^ns^ |
| NP | 23.43±1.25b^*^ | 473.84±1.02a^ns^ | 1.18±0.12a^ns^ | 38.44±4.87a^ns^ | 2686.44±198.55ab^ns^ | 25.64±3.05ab^ns^ | 37.18±2.57b^ns^ | 7.83±0.62a^ns^ |
| CdH | 20.37±0.70B | 489.78±4.72A | 0.89±0.04B | 50.89±2.45AB | 2526.00±187.36B | 19.67±1.10AB | 41.23±2.64A | 7.14±0.16AB |
| CdHN | 27.92±0.27A | 464.95±2.58C | 1.38±0.03A | 38.72±2.07B | 4008.67±79.14A | 25.25±1.65A | 40.46±4.39A | 8.26±0.07A |
| CdHP | 22.00±1.51B | 492.74±2.17A | 1.05±0.09B | 60.29±9.86A | 3002.27±439.78B | 24.27±1.72AB | 42.95±6.94A | 6.48±0.16B |
| CdHNP | 27.97±0.55A | 476.43±0.54B | 0.97±0.07B | 44.52±2.15AB | 2744.16±81.46B | 18.95±2.23B | 45.45±3.32A | 8.18±0.65A |

Notes: Values are means ± SE (n=3). Different letters on the bars indicate significant differences between the treatments (p<0.05) obtained via Duncan's test. Lowercase letters indicate significant differences between different treatments under cadmium free stress. Capital letters indicate significant differences between different treatments under cadmium stress. Asterisks denote statistically significant differences between the two Cd levels at p<0.05 according to independent-samples t-test (*: p<0.05; **: p<0.01; ns: p>0.05).

**Table S2. The change in total nitrogen, total carbon and various important nutrients in the second order roots of *Phoebe zhennan* among different treatments**.

| **Treatment/2^nd^ root** | **TN (g/kg)** | **TC (g/kg)** | **K (g/kg)** | **Cu (mg/kg)** | **Fe (mg/kg)** | **Mn (mg/kg)** | **Zn (mg/kg)** | **Ca (g/kg)** |
| --- | --- | --- | --- | --- | --- | --- | --- | --- |
| CK | 17.74±0.39b^ns^ | 466.67±4.99a^ns^ | 1.46±0.10a^*^ | 52.65±6.06a^ns^ | 1768.05±404.50b^**^ | 36.55±2.83a^*^ | 57.94±10.08a^ns^ | 5.59±0.39b^ns^ |
| N | 26.99±1.03a^ns^ | 466.80±0.16a^**^ | 1.42±0.10ab^ns^ | 32.69±1.09b^ns^ | 2558.00±94.30a^**^ | 29.62±2.15ab^**^ | 33.71±7.97b^ns^ | 6.70±0.41ab^ns^ |
| P | 16.78±1.53b^ns^ | 473.88±1.20a^ns^ | 1.05±0.03c^ns^ | 39.12±1.64b^*^ | 2335.73±145.52a^ns^ | 33.91±2.01a^*^ | 29.22±0.86b^*^ | 7.65±0.45a^*^ |
| NP | 23.84±1.65a^ns^ | 469.55±1.73a^ns^ | 1.10±0.14bc^ns^ | 27.70±4.27b^*^ | 2240.44±140.61a^**^ | 22.72±1.51b^ns^ | 37.38±2.88ab^ns^ | 7.12±0.34a^ns^ |
| CdH | 19.34±0.69B | 489.85±10.12A | 1.00±0.02B | 54.89±2.15A | 2470.56±215.56BC | 22.78±1.44B | 48.74±0.44AB | 6.48±0.33B |
| CdHN | 24.45±0.05A | 450.60±2.71B | 1.41±0.12A | 46.65±9.22A | 4603.32±267.98A | 40.88±0.42A | 38.74±4.68B | 7.26±0.05A |
| CdHP | 19.69±1.74B | 482.20±3.09A | 0.94±0.06B | 50.33±3.17A | 2788.05±491.42C | 24.18±1.59B | 36.51±1.65B | 5.83±0.17B |
| CdHNP | 24.17±0.33A | 474.33±1.60A | 0.89±0.02B | 48.76±2.27A | 3008.55±25.55B | 20.20±0.37B | 53.77±7.04A | 7.35±0.26A |

Notes: Values are means ± SE (n=3). Different letters on the bars indicate significant differences between the treatments (p<0.05) obtained via Duncan's test. Lowercase letters indicate significant differences between different treatments under cadmium free stress. Capital letters indicate significant differences between different treatments under cadmium stress. Asterisks denote statistically significant differences between the two Cd levels at p<0.05 according to independent-samples t-test (*: p<0.05; **: p<0.01; ns: p>0.05).

**Table S3. The change in total nitrogen, total carbon and various important nutrients in the third order roots of *Phoebe zhennan* among different treatments**.

| **Treatment/3^rd^ root** | **TN (g/kg)** | **TC (g/kg)** | **K (g/kg)** | **Cu (mg/kg)** | **Fe (mg/kg)** | **Mn (mg/kg)** | **Zn (mg/kg)** | **Ca (g/kg)** |
| --- | --- | --- | --- | --- | --- | --- | --- | --- |
| CK | 15.58±0.72b^ns^ | 465.03±4.20b^ns^ | 1.22±0.02a^ns^ | 46.38±7.17a^ns^ | 1473.76±135.02b^**^ | 44.74±0.75a^**^ | 47.61±12.58a^ns^ | 5.09±0.17c^**^ |
| N | 24.69±0.94a^*^ | 473.64±2.12ab^ns^ | 1.10±0.03ab^ns^ | 34.81±0.65a^ns^ | 2571.30±148.02a^**^ | 33.11±2.29b^ns^ | 38.72±6.70a^ns^ | 6.92±0.27a^ns^ |
| P | 16.90±2.10b^ns^ | 476.45±2.23a^**^ | 0.85±0.05c^ns^ | 37.47±1.26a^*^ | 2505.12±134.59a^ns^ | 34.76±2.21b^ns^ | 33.07±0.33a^ns^ | 6.49±0.20ab^*^ |
| NP | 18.75±0.26b^**^ | 478.72±1.14a^ns^ | 1.04±0.07b^ns^ | 34.84±8.68a^ns^ | 2553.52±454.46a^ns^ | 26.00±2.57c^ns^ | 28.90±3.99a^*^ | 5.71±0.41bc^*^ |
| CdH | 17.16±0.35B | 480.12±0.47B | 0.99±0.08AB | 51.70±3.15A | 3100.23±245.86B | 30.37±2.83AB | 52.20±2.66A | 6.90±0.07AB |
| CdHN | 21.10±0.27A | 470.99±1.90C | 1.15±0.09A | 47.90±9.33A | 4404.63±116.24A | 36.68±3.24A | 41.99±4.42A | 6.39±0.22B |
| CdHP | 17.02±1.26B | 489.81±1.53A | 0.77±0.04B | 48.92±2.32A | 2454.21±56.21C | 28.61±1.37BC | 52.36±8.83A | 5.26±0.22C |
| CdHNP | 21.26±0.19A | 478.77±1.85B | 0.80±0.03B | 45.18±1.04A | 2766.09±124.31BC | 21.75±0.59C | 55.06±1.44A | 7.34±0.13A |

Notes: Values are means ± SE (n=3). Different letters on the bars indicate significant differences between the treatments (p<0.05) obtained via Duncan's test. Lowercase letters indicate significant differences between different treatments under cadmium free stress. Capital letters indicate significant differences between different treatments under cadmium stress. Asterisks denote statistically significant differences between the two Cd levels at p<0.05 according to independent-samples t-test (*: p<0.05; **: p<0.01; ns: p>0.05).

**Table S4. The change in total nitrogen, total carbon and various important nutrients in the stem of *Phoebe zhennan* among different treatments**.

| **Treatment/stem** | | **TN (g/kg)** | **TC (g/kg)** | **K (g/kg)** | **Cu (mg/kg)** | **Fe (mg/kg)** | **Mn (mg/kg)** | **Zn (mg/kg)** | **Ca (g/kg)** |
| --- | --- | --- | --- | --- | --- | --- | --- | --- | --- |
| CK | 8.10±0.61b^**^ | | 476.79±4.60a^ns^ | 4.02±0.15b^ns^ | 11.70±0.88a^ns^ | 112.90±0.33b^**^ | 50.98±3.56ab^ns^ | 12.67±1.60a^ns^ | 9.88±0.44a^ns^ |
| N | 13.16±0.63a^ns^ | | 483.39±4.35a^ns^ | 3.26±0.21b^ns^ | 11.95±2.19a^ns^ | 118.07±5.12ab^ns^ | 42.77±2.33b^ns^ | 9.58±0.49a^ns^ | 9.86±0.35a^ns^ |
| P | 8.02±1.07b^ns^ | | 475.76±3.64a^ns^ | 5.58±0.69a^ns^ | 11.77±1.33a^ns^ | 116.61±4.78b^*^ | 69.02±9.44a^ns^ | 14.24±2.88a^ns^ | 9.24±0.11a^ns^ |
| NP | 13.80±1.00a^ns^ | | 480.45±1.35a^ns^ | 4.46±0.12ab^ns^ | 10.11±1.56a^ns^ | 139.82±12.03a^*^ | 62.85±6.49ab^ns^ | 11.13±0.86a^ns^ | 8.97±0.29a^ns^ |
| CdH | 11.97±0.13B | | 477.00±0.64A | 4.46±0.78A | 10.26±0.92A | 170.94±6.66AB | 50.38±3.76AB | 13.77±1.51A | 10.24±0.04A |
| CdHN | 14.35±0.34A | | 483.62±4.54A | 4.29±0.48A | 10.26±0.99A | 140.38±7.89B | 36.81±3.93B | 12.20±1.17A | 9.09±0.34B |
| CdHP | 9.94±0.94C | | 476.02±3.27A | 5.22±0.48A | 10.37±0.97A | 228.37±37.55A | 71.85±10.76A | 14.42±0.94A | 8.81±0.36B |
| CdHNP | 15.19±0.20A | | 480.85±1.42A | 4.87±0.20A | 11.02±0.13A | 179.82±7.58AB | 58.42±4.21AB | 13.40±0.27A | 9.52±0.21AB |

Notes: Values are means ± SE (n=3). Different letters on the bars indicate significant differences between the treatments (p<0.05) obtained via Duncan's test. Lowercase letters indicate significant differences between different treatments under cadmium free stress. Capital letters indicate significant differences between different treatments under cadmium stress. Asterisks denote statistically significant differences between the two Cd levels at p<0.05 according to independent-samples t-test (*: p<0.05; **: p<0.01; ns: p>0.05).

**Table S5. The change in total nitrogen, total carbon and various important nutrients in the leaves of *Phoebe zhennan* among different treatments**.

| **Treatment/leaf** | **TN (g/kg)** | **TC (g/kg)** | **K (g/kg)** | **Cu (mg/kg)** | **Fe (mg/kg)** | **Mn (mg/kg)** | **Zn (mg/kg)** | **Ca (g/kg)** |
| --- | --- | --- | --- | --- | --- | --- | --- | --- |
| CK | 21.09±0.14ab^ns^ | 490.71±0.32a^*^ | 5.82±0.41a^ns^ | 8.04±0.15a^**^ | 241.19±16.14b^*^ | 122.26±6.66b^ns^ | 26.32±2.91ab^ns^ | 22.68±0.75b^*^ |
| N | 21.73±0.56ab^ns^ | 481.64±0.68b^ns^ | 5.72±0.17ab^ns^ | 8.05±0.13a^ns^ | 448.68±65.69a^*^ | 187.21±4.90a^**^ | 29.11±0.56a^ns^ | 30.94±1.28a^ns^ |
| P | 19.96±1.61b^ns^ | 492.78±3.03a^ns^ | 5.17±0.15ab^ns^ | 7.07±0.11b^ns^ | 324.79±23.95b^ns^ | 125.54±21.73b^ns^ | 21.45±0.54b^*^ | 23.92±1.44b^ns^ |
| NP | 23.96±0.25a^ns^ | 491.35±0.72a^ns^ | 4.81±0.30b^ns^ | 6.53±0.10c^ns^ | 259.34±9.26b^ns^ | 154.01±16.06ab^ns^ | 21.59±1.23b^*^ | 27.87±1.18a^ns^ |
| CdH | 20.62±1.03B | 488.77±0.47B | 5.50±0.16A | 6.35±0.12A | 365.08±20.60A | 142.80±10.61A | 25.46±2.80A | 28.89±1.92A |
| CdHN | 22.16±0.52AB | 478.97±1.66C | 5.95±1.42A | 6.26±0.65A | 244.56±18.34C | 113.48±3.77A | 22.44±3.83A | 29.17±0.53A |
| CdHP | 17.00±0.89C | 492.98±1.18A | 5.10±0.17A | 6.34±0.27A | 337.70±23.36AB | 141.44±8.03A | 28.08±1.44A | 20.88±2.04B |
| CdHNP | 24.43±0.49A | 491.76±0.79AB | 4.34±0.30A | 6.51±0.05A | 289.76±8.15BC | 101.76±50.22A | 28.36±1.85A | 28.18±0.55A |

Notes: Values are means ± SE (n=3). Different letters on the bars indicate significant differences between the treatments (p<0.05) obtained via Duncan's test. Lowercase letters indicate significant differences between different treatments under cadmium free stress. Capital letters indicate significant differences between different treatments under cadmium stress. Asterisks denote statistically significant differences between the two Cd levels at p<0.05 according to independent-samples t-test (*: p<0.05; **: p<0.01; ns: p>0.05).

**Table S6. The change in plant height, ground diameter, leaf dry weight, stem** **dry weight, root dry weight, aboveground biomass, and the total biomass of *Phoebe zhennan* among different treatments**.

| **Treatment** | **IPH (cm)** | **IGD (cm)** | **LDW**  **(g·plant-1·DW)** | **SDW**  **(g·plant-1·DW)** | **RDW**  **(g·plant-1·DW)** | **AB**  **(g·plant-1·DW)** | **TB**  **(g·plant-1·DW)** |
| --- | --- | --- | --- | --- | --- | --- | --- |
| CK | 18.00±3.46bc^ns^ | 4.68±0.35bc^ns^ | 13.22±0.67b^**^ | 26.60±1.01a^**^ | 17.09±1.92a^ns^ | 39.81±1.65ab^**^ | 56.90±3.55ab^**^ |
| N | 12.33±1.76c^ns^ | 3.75±0.45c^ns^ | 9.20±0.74c^ns^ | 18.91±1.23b^**^ | 5.51±0.94b^ns^ | 28.11±1.89c^**^ | 33.62±2.76c^**^ |
| P | 23.00±3.51ab^ns^ | 5.78±0.17ab^ns^ | 16.76±1.52a^ns^ | 31.58±2.61a^**^ | 17.61±3.51a^ns^ | 48.34±4.12a^*^ | 65.94±7.06a^ns^ |
| NP | 28.33±2.33a^ns^ | 6.01±0.46a^*^ | 15.40±0.96ab^**^ | 15.28±1.96b^*^ | 13.63±1.05a^ns^ | 30.68±2.90bc^*^ | 44.31±3.94bc^ns^ |
| CdH | 14.67±0.88A | 3.52±0.57B | 8.42±0.55BC | 8.77±1.19B | 11.07±2.04BC | 17.19±1.72B | 28.26±3.63B |
| CdHN | 6.00±1.73B | 3.73±0.42B | 6.71±0.87C | 5.58±0.98C | 6.55±1.37C | 9.59±1.71C | 14.35±2.98C |
| CdHP | 19.67±4.70A | 5.85±0.02A | 14.74±0.23A | 15.04±0.48A | 16.36±1.43A | 29.66±0.82A | 46.17±1.68A |
| CdHNP | 21.67±1.45A | 4.39±0.11B | 9.95±0.08B | 8.21±0.32BC | 11.73±1.01AB | 17.87±0.37B | 32.44±4.99B |

Notes: Values are means ± SE (n=3). Different letters on the bars indicate significant differences between the treatments (p<0.05) obtained via Duncan's test. Lowercase letters indicate significant differences between different treatments under cadmium free stress. Capital letters indicate significant differences between different treatments under cadmium stress. Asterisks denote statistically significant differences between the two Cd levels at p<0.05 according to independent-samples t-test (*: p<0.05; **: p<0.01; ns: p>0.05).

**Table S7. The change in root length, average root diameter, root surface, and root volume of *Phoebe zhennan* among different treatments.**

| Treatment | RL (cm) | ARD (mm) | RS (cm2) | RV (cm3) |
| --- | --- | --- | --- | --- |
| CK | 7814.39±851.99a^ns^ | 6.22±0.61ab^ns^ | 1946.51±114.94a^ns^ | 39.23±1.91a^ns^ |
| N | 3242.86±974.48b^ns^ | 3.63±0.34c^ns^ | 1122.53±225.79b^ns^ | 18.84±4.11b^ns^ |
| P | 9869.16±1121.99a^ns^ | 4.91±0.55bc^*^ | 2186.21±299.77a^ns^ | 29.21±3.31a^*^ |
| NP | 8701.40±338.52a^**^ | 6.70±0.22a^**^ | 1902.10±85.56a^*^ | 30.36±1.91a^ns^ |
| CdH | 7403.87±363.57AB | 6.98±0.47A | 1766.24±96.96B | 33.63±2.02B |
| CdHN | 2381.98±715.13C | 3.64±0.52C | 919.99±130.31C | 18.04±1.56C |
| CdHP | 8874.18±752.24A | 7.35±0.17A | 2076.09±88.24A | 43.59±2.66A |
| CdHNP | 6577.83±182.97B | 5.41±0.02B | 1595.17±39.57B | 30.85±0.69B |

Notes: RL; root length, ARD; average root diameter, RS; root surface, RV; root volume. Values are means ± SE (n=3). Different letters on the bars indicate significant differences between the treatments (p<0.05) obtained via Duncan's test. Lowercase letters indicate significant differences between different treatments under cadmium free stress. Capital letters indicate significant differences between different treatments under cadmium stress. Asterisks denote statistically significant differences between the two Cd levels at p<0.05 according to independent-samples t-test (*: p<0.05; **: p<0.01; ns: p>0.05).

**Table S8. Cd levels in different organs of *Phoebe zhennan* among different treatments.**

| **Treatment** | **Leaf Cd (mg/kg)** | **stem Cd**  **(mg/kg)** | **1st root Cd (mg/kg)** | **2nd root Cd (mg/kg)** | **3rd root Cd (mg/kg)** |
| --- | --- | --- | --- | --- | --- |
| CK | 0.22±0.01a^**^ | 0.39±0.01c^**^ | 1.66±0.06b^**^ | 2.10±0.09b^**^ | 1.41±0.10b^**^ |
| N | 0.24±0.05a^**^ | 0.89±0.04a^**^ | 2.26±0.07ab^**^ | 2.18±0.25b^**^ | 2.47±0.52a^**^ |
| P | 0.17±0.01a^**^ | 0.34±0.01c^**^ | 1.92±0.27ab^**^ | 1.41±0.08c^**^ | 1.41±0.04b^**^ |
| NP | 0.19±0.01a^**^ | 0.54±0.04b^**^ | 2.90±0.53a^**^ | 2.93±0.23a^**^ | 2.00±0.29ab^**^ |
| CdH | 2.49±0.24B | 28.43±0.59BC | 95.25±8.43A | 101.70±10.65A | 95.82±11.45A |
| CdHN | 26.23±7.45A | 50.14±6.54A | 78.67±3.48AB | 75.10±6.71B | 62.60±6.03B |
| CdHP | 3.90±1.05B | 20.20±2.09C | 70.24±7.56B | 65.25±2.95B | 61.04±4.01B |
| CdHNP | 2.26±0.41B | 35.26±3.47AB | 77.26±2.60AB | 62.95±2.67B | 55.88±4.45B |

Notes: Values are means ± SE (n=3). Different letters on the bars indicate significant differences between the treatments (p<0.05) obtained via Duncan's test. Lowercase letters indicate significant differences between different treatments under cadmium free stress. Capital letters indicate significant differences between different treatments under cadmium stress. Asterisks denote statistically significant differences between the two Cd levels at p<0.05 according to independent-samples t-test (*: p<0.05; **: p<0.01; ns: p>0.05).

**Table S9. Fuzzy differential subordination analysis of N, P, and NP addition under Cd stress.**

| **Treatment** | **CK** | **N** | **P** | **NP** | **CdH** | **CdHN** | **CdHP** | **CdHNP** |
| --- | --- | --- | --- | --- | --- | --- | --- | --- |
| IPH | 0.54 | 0.28 | 0.76 | 1.00 | 0.39 | 0.00 | 0.61 | 0.70 |
| IGD | 0.47 | 0.09 | 0.91 | 1.00 | 0.00 | 0.09 | 0.94 | 0.35 |
| LDW | 0.65 | 0.25 | 1.00 | 0.86 | 0.17 | 0.00 | 0.80 | 0.32 |
| SDW | 0.81 | 0.51 | 1.00 | 0.37 | 0.12 | 0.00 | 0.36 | 0.10 |
| RDW | 0.96 | 0.00 | 1.00 | 0.67 | 0.46 | 0.09 | 0.90 | 0.51 |
| AB | 0.78 | 0.48 | 1.00 | 0.54 | 0.20 | 0.00 | 0.52 | 0.21 |
| TB | 0.82 | 0.37 | 1.00 | 0.58 | 0.27 | 0.00 | 0.62 | 0.35 |
| 1st RDW | 0.58 | 0.00 | 1.00 | 0.73 | 0.69 | 0.11 | 0.85 | 0.56 |
| 2nd RDW | 0.72 | 0.00 | 1.00 | 0.62 | 0.48 | 0.02 | 0.92 | 0.28 |
| 3rd RDW | 1.00 | 0.00 | 0.97 | 0.62 | 0.40 | 0.00 | 0.84 | 0.29 |
| RL | 0.73 | 0.11 | 1.00 | 0.84 | 0.67 | 0.00 | 0.87 | 0.56 |
| RS | 0.81 | 0.16 | 1.00 | 0.78 | 0.67 | 0.00 | 0.91 | 0.53 |
| ARD | 0.69 | 0.00 | 0.34 | 0.82 | 0.90 | 0.00 | 1.00 | 0.48 |
| RV | 0.83 | 0.03 | 0.44 | 0.48 | 0.61 | 0.00 | 1.00 | 0.50 |
| Chla | 0.61 | 0.00 | 0.65 | 1.00 | 0.44 | 0.64 | 0.74 | 0.83 |
| Chlb | 0.63 | 0.00 | 0.58 | 0.89 | 0.53 | 0.64 | 1.00 | 0.81 |
| Caro | 0.49 | 0.00 | 0.72 | 1.00 | 0.48 | 0.63 | 0.70 | 0.83 |
| TChl | 0.62 | 0.00 | 0.65 | 1.00 | 0.46 | 0.65 | 0.79 | 0.84 |
| Pn | 0.76 | 0.56 | 0.85 | 1.00 | 0.20 | 0.00 | 0.26 | 0.50 |
| Gs | 0.71 | 0.57 | 1.00 | 0.86 | 0.29 | 0.00 | 0.43 | 0.50 |
| Ci | 0.50 | 0.37 | 0.76 | 0.02 | 0.64 | 0.00 | 1.00 | 0.59 |
| Tr | 0.19 | 0.24 | 0.56 | 1.00 | 0.18 | 0.00 | 0.22 | 0.60 |
| O2·– | 0.17 | 0.24 | 0.00 | 0.09 | 1.00 | 0.77 | 0.33 | 0.42 |
| RC(%) | 0.96 | 1.00 | 1.00 | 0.83 | 0.00 | 0.06 | 0.19 | 0.00 |
| MDA | 0.95 | 0.77 | 1.00 | 0.78 | 0.59 | 0.00 | 0.59 | 0.66 |
| Pro | 0.70 | 0.73 | 0.93 | 0.73 | 0.00 | 0.15 | 1.00 | 0.26 |
| SP | 1.00 | 0.70 | 0.44 | 0.37 | 0.00 | 0.26 | 0.45 | 0.44 |
| GSH | 0.92 | 0.83 | 1.00 | 0.83 | 0.56 | 0.00 | 0.84 | 0.75 |
| POD | 0.38 | 0.95 | 0.82 | 1.00 | 0.45 | 0.00 | 0.30 | 0.63 |
| SOD | 0.41 | 0.19 | 0.60 | 1.00 | 0.00 | 0.20 | 0.40 | 0.28 |
| CAT | 0.99 | 0.00 | 1.00 | 0.85 | 0.28 | 0.29 | 0.89 | 0.40 |
| APX | 0.46 | 0.55 | 1.00 | 0.36 | 0.00 | 0.02 | 0.75 | 0.15 |
| leaf Cd (mg/kg) | 0.00 | 0.00 | 0.00 | 0.00 | 0.09 | 1.00 | 0.14 | 0.08 |
| Stem Cd (mg/kg) | 0.00 | 0.01 | 0.00 | 0.00 | 0.56 | 1.00 | 0.40 | 0.70 |
| 1st root (Cd mg/kg) | 0.00 | 0.01 | 0.00 | 0.01 | 1.00 | 0.82 | 0.73 | 0.81 |
| 2nd root (Cd mg/kg) | 0.01 | 0.01 | 0.00 | 0.02 | 1.00 | 0.73 | 0.64 | 0.61 |
| 3st root (Cd mg/kg) | 0.00 | 0.01 | 0.00 | 0.01 | 1.00 | 0.65 | 0.63 | 0.58 |
| Mean | 0.59 | 0.27 | 0.70 | 0.64 | 0.43 | 0.24 | 0.66 | 0.49 |


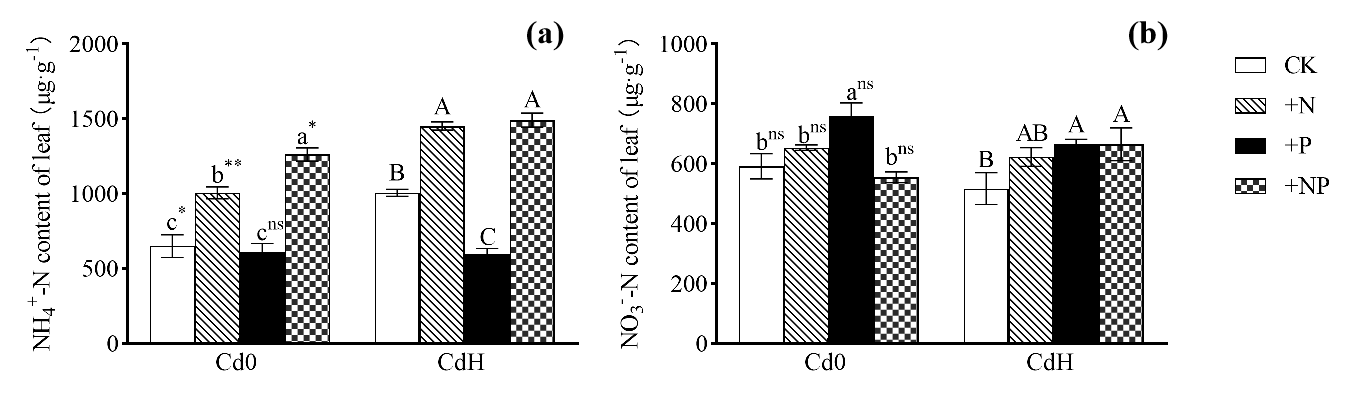


**Supplementary Figure1. Preferences for different forms of nitrogen in the leaves.** (a) NH_4_^+^-N content in the leaves. (b) NO_3_^-^-N content in the leaves. Notes: Values are means ± SE (n=3). Different letters on the bars indicate significant differences between the treatments (p<0.05) obtained via Duncan's test. Lowercase letters indicate significant differences between different treatments under cadmium free stress. Capital letters indicate significant differences between different treatments under cadmium stress. Asterisks denote statistically significant differences between the two Cd levels at p<0.05 according to independent-samples t-test (*: p<0.05; **: p<0.01; ns: p>0.05).
